# A systematic review with meta-analysis on the relation between acute stress, alcohol consumption and cortisol levels in individuals with a personal, familial or no alcohol use disorder

**DOI:** 10.1038/s41398-025-03641-8

**Published:** 2025-10-20

**Authors:** Lisa J. Weckesser, Maximilian Pilhatsch, Markus Muehlhan, Tanja Endraß, Robert Miller

**Affiliations:** 1 Institute of Clinical Psychology and Psychotherapy, Technische Universität Dresden, Dresden, Germany; 2 Department of Psychiatry and Psychotherapy, Faculty of Medicine, Technische Universität Dresden, Dresden, Germany; 3 Partner Site Leipzig / Dresden, German Center for Child and Adolescent Health (DZKJ), Göttingen, Germany; 4 Department of Psychology / ICAN Institute of Cognitive and Affective Neuroscience, MSH Medical School Hamburg, Hamburg, Germany; 5 Department of Psychological Methods, Psychologische Hochschule Berlin, Berlin, Germany

**Keywords:** Addiction, Human behaviour

## Abstract

The self-medication hypothesis posits that alcohol is consumed to cope with stress, but corresponding evidence (incl. its impact on physiological stress markers) is heterogeneous. Thus, this systematic review examined the relationship between acute stress, acute alcohol consumption, and cortisol levels in individuals with varying alcohol use disorder (AUD) statuses. Following PRISMA guidelines, records from PubMed/Medline, Web of Science, and PsycInfo up to 16/05/2025 were analysed. We included 53 experimental and 31 observational studies that reported alcohol consumption volume after stress versus control conditions or measured cortisol *recovery* (C_Min_) and *reactivity* (C_MaxMin_) following alcohol intake with or without concurrent stressor exposure. Samples were classified by AUD status (active AUD, previously diagnosed but abstinent, or no AUD). Additionally, the moderating effect of family history of AUD was investigated. Regarding effects of acute stress on alcohol consumption, stressor exposure only increased alcohol consumption when small-study effects were not accounted for (g_REE _= 0.25), but this effect disappeared with adjustments (g_PEESE _= 0.02). Acute alcohol consumption was associated with delayed cortisol *recovery* (C_Min_↑; g_REE_ = 0.15), especially in active AUD individuals (g_REE _= 0.46) and those with a familial history of AUD (g_REE _= 0.16). However, alcohol intake did not significantly affect cortisol reactivity (β = −0.01), although contrasting effects were seen in active AUD individuals (C_MaxMin_↑) and those with familial AUD histories (C_MaxMin_↓). In conclusion, acute stress does not increase alcohol consumption, and alcohol consumption does not regulate cortisol peak responses. Since alcohol consumption altered cortisol recovery, minimal cortisol levels may be a sensitive marker of alcohol-related change in adrenal functioning contributing to AUD progression.

## Introduction

Since the 1950s, the tension-reduction and self-medication hypotheses (SMH) postulate that individuals consume alcohol to alleviate negative feelings of anxiety or tension or enhance sedation [1–4]. Under stress, alcohol is supposedly consumed to reduce negative feelings of being stressed, helpless or insecure (or induce positive feelings of stimulation or liking) [5–7]. Occasionally occurring, such alcohol consumption helps terminating physiological and subjective stress responses and can thus be seen as an adaptive coping mechanism [2, 8]. When alcohol is consumed for those regulatory properties, the SMH suggest a general increase in alcohol consumption under stress, regardless of the specific (stress-related) feeling that requires regulation or the individual’s history of alcohol use disorder (AUD) [9–11].

Empirical findings support some assumptions made by the SMH, for example that ego-threatening psychosocial stress increases alcohol consumption [12–14]. Similar increases are observed with non-alcoholic or placebo beverages, suggesting influences beyond the pharmacological and regulatory effects of alcohol [15, 16]. However, acute alcohol consumption changes stress indicators (e.g., heart rate or cortisol), similarly to acute stress [8, 17, 18]. Since alcohol cannot mimic and alleviate such stress-induced changes at the same time scale, the relation between alcohol consumption and stress is arguably more complex than initially suggested by the SMH [3, 19]. To address this complexity, research has focused on cortisol, the principal steroid hormone synthesized by the hypothalamic-pituitary-adrenal (HPA) axis [7, 19, 20]. As a stress-sensitive biomarker, cortisol provides one out of several objective means to investigate how alcohol modulates the stress response [7, 19]. Consequently, the moderating contribution of cortisol has been examined to untangle the relation between acute stress and harmful alcohol consumption, offering a critical translational link to understanding risk and consequences of stress in the progression of AUD [17, 19, 20].

Based on these investigations, the relationship between stress, cortisol and alcohol can be described by several competing hypotheses: If alcohol is consumed to alleviate anxiety and tension, or to induce sedation, stressor exposure should increase both the volume of alcoholic beverage consumed (A_Vol_) and the amount of pharmacologically effective pure alcohol ingested (A_Mass_) [8, 15]. If this stress effect is attributable to alcohol’s regulatory, tension-reducing [21] or sedation-enhancing [4] properties, then alcohol consumption, especially under stress, should reduce resulting cortisol, thereby mitigating any alcohol- and stress-induced cortisol increases [18, 22]. Such increases are best described by the differences between maximal and minimal cortisol levels (C_MaxMin_) or cortisol *reactivity* [23], which should be lower under acute alcohol consumption as compared to control. Conversely, if alcohol acts as an independent stressor [17, 19, 20], alcohol consumption, alone or combined with stressor exposure, should increase cortisol, resulting in higher alcohol- and stress-induced cortisol reactivity (C_MaxMin_). To comprehensively characterize stress-related cortisol secretion, the return of elevated cortisol to a steady state (“baseline”) level should further be described by minimal cortisol levels (C_Min_), where higher C_Min_ reflects a slower return to “baseline” and poorer regulatory capacity [23]. Since cortisol and occasionally consumed alcohol are primarily metabolized through distinct pathways [24], C_Min_ should not be systematically affected by singular acute alcohol consumption or by its interaction with stressor exposure [23, 25].

Regarding repeated alcohol consumption, previous findings suggest an increase in mean basal cortisol levels but decrease in cortisol *reactivity* to additional stressor exposure (supposedly an adaptive response to repeated alcohol consumption; cf. “Research and translational implications”) [26–28]. Consequently, individuals repeatedly consuming alcohol should exhibit reduced cortisol *recovery* (higher C_Min_) and reduced cortisol *reactivity* (lower C_MaxMin_) in response to alcohol and stressor exposure. AUD is highly heritable, with a positive family history being among its most significant risk factor, even in absence of current drinking [29, 30]. Thus, if such change precedes the onset of an active AUD drinking pattern and/or adapts following its cessation, then a positive family history of alcohol consumption and/or abstinence from a previous AUD drinking pattern should moderate said effects of alcohol consumption and stressor exposure on cortisol *reactivity* (C_MaxMin_) and *recovery* (C_Min_).

To evaluate these hypotheses, we systematically reviewed and aggregated primary studies investigating the relation between alcohol consumption, stressor exposure and their impact on cortisol. Our systematic review with meta-analysis aimed to estimate (1) the effect of stressor exposure on alcohol consumption, and (2) the effect of alcohol consumption, with or without concurrent stressor exposure, on cortisol. We also evaluated whether heterogeneity in those effects was associated with the samples’ alcohol consumption history, including their personal history of alcohol use disorder classifying individuals as currently drinking with an AUD (AUD+), previously diagnosed but now abstinent (AUD±), or without an AUD diagnosis (AUD-), their family history of AUD (FH status), and the type of stress-induction protocol used (ranging from imagery, social-evaluative, cognitive, physiologic to pharmacologic stress-induction types). All of these variables have previously been considered relevant to the relation between acute stress, alcohol consumption and cortisol [19, 26, 31, 32].

Despite decades of research, empirical findings on the interplay between acute stress, alcohol consumption, and cortisol remain inconsistent, with primary studies often yielding conflicting results [15, 18, 31]. These inconsistencies have complicated efforts to validate or refute prominent theoretical frameworks such as the SMH and its extensions (cf. “Research and translational implications”) [3, 19]. To date, no comprehensive meta-analysis has quantitatively synthesized this literature to resolve these conflicts and evaluate the role of key moderators, including personal and family history of AUD. This systematic review and meta-analysis was therefore conducted to address this critical gap by providing the first aggregated estimates of these effects and their moderators.

## Methods

### Search strategy

**Fig. 1:**
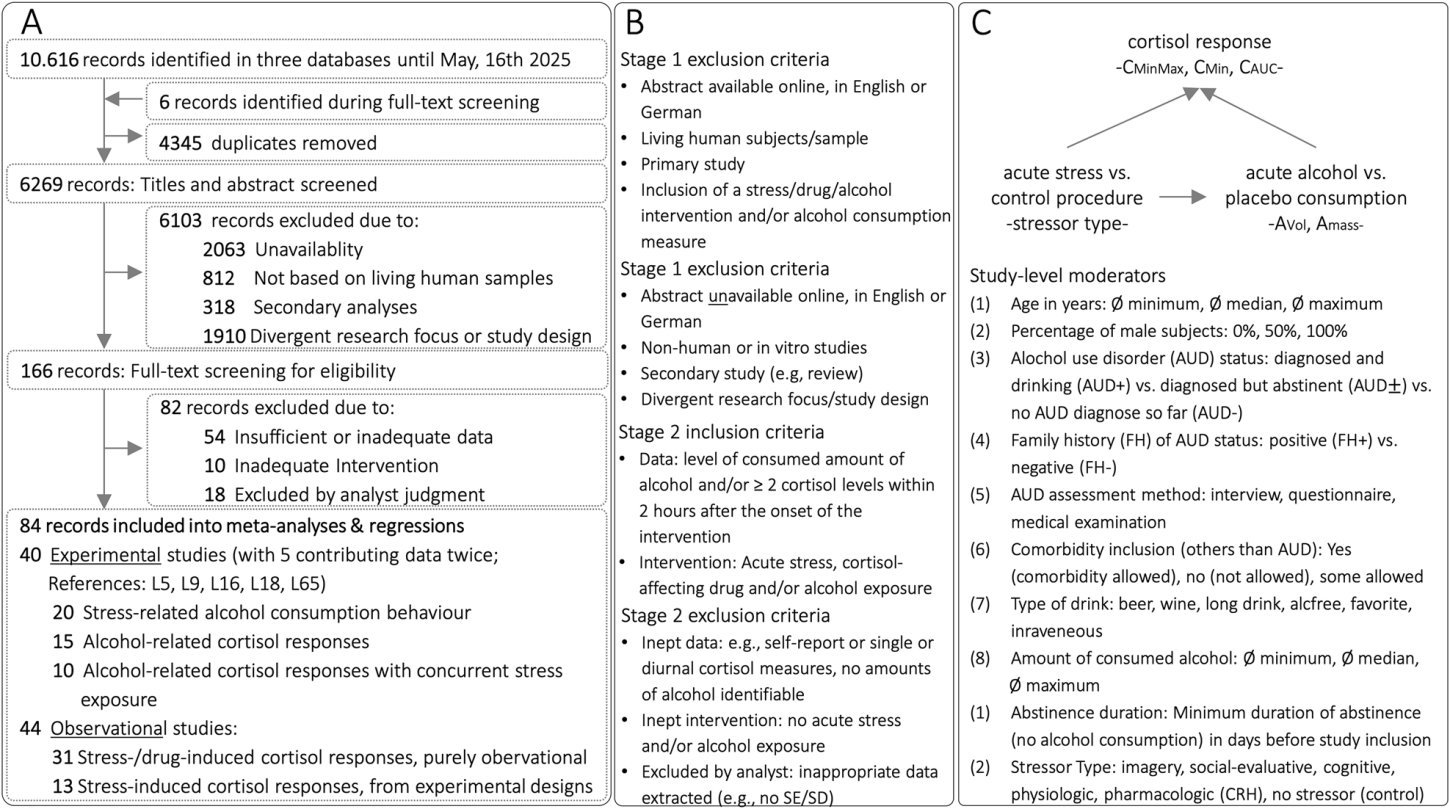
Study Design. **A** PRISMA flow diagram depicting the two systematic literature searches conducted in *PubMed/Medline*, *Web of Science*, and *PsycInfo* databases (the initial search included records up to July 31, 2023, and a second search during revision including records up to May 16, 2025). The study’s (**B**) inclusion and exclusion criteria, and (**C**) analytical model along with potential study-level moderators that may affect the analyzed effect size estimates. C_MaxMin_=cortisol reactivity as difference between maximum and minimum levels, C_Min_=cortisol recovery as minimum levels, C_AUC_=cortisol concentration time course; A_Vol_=volume of consumed alcohol in ml, A_mass_=consumed mass of pure alcohol in g. CRH=Corticotropin-releasing hormone.

In accordance with the PRISMA guidelines, we conducted a comprehensive search for primary studies examining the relation between acute alcohol consumption, acute stress, and cortisol [33]. The search encompassed entries available in the *PubMed/Medline*, *Web of Science*, and *PsycInfo* databases up to July 31, 2023 and was updated during the (external) review up to May 16, 2025 using the R package *litsearchr* [34]. Our search strategy was pre-registered on PROSPERO (ID: CRD42023441163) and employed a combination of the search terms “stress or stressor or cortisol or HPA axis or corticotropin or CRH or hypothalamus-pituitary-adrenal axis or provocation or reactivity” and “alcohol or abstinence or ethanol or alcohol-dependent or alcoholic or alcoholics or withdrawal or relapse or drinking or consumption” but excluded “mice or mouse or rat or rats or monkey or rodent or child or children or oxidative” in article titles (see Fig. 1A). The identification of individual words within this search term was permitted (e.g. “alcohol” also included “alcohol misuse”).

### Inclusion criteria

We selected primary studies involving human participants with a mean age of at least 18 years to minimize putative age-related differences in cortisol responses and alcohol effects, and divergent developmental and life-transition factors that are beyond the scope of the present review [35–38]. Eligible studies were required to be available online in English or German and to report data on both alcohol consumption behaviour and either stressor exposure and/or cortisol levels in texts, figures, or tables. This encompassed the identification of experimental and observational studies investigating (i) the effect of stressor exposure on alcohol consumption behaviour (as compared to a control exposure), (ii) the effect of alcohol compared to placebo (non-alcoholic beverage) consumption on cortisol, across at least two sampling occasions (one before and one after the respective exposures), and (iii) the moderation of these effects by the samples’ AUD status and FH status (see “Data extraction and preparation”). For reasons of conciseness, we focused on those studies assessing alcohol consumption in response to stress and cortisol response to stress or alcohol consumption within 2 h of the interventions’ start. That is, studies exclusively reporting diurnal cortisol levels were not analyzed for this report.

### Exclusion criteria

Entries from the databases were excluded if they reported secondary (including reviews, meta-analyses, guidelines, comments), insufficient data (e.g. meeting abstracts, or case studies), or incompatible outcome measures (e.g., self-report measures of alcohol consumption or subjective stress). Specifically, we excluded studies that used self-report as the primary outcome measure for acute alcohol consumption (e.g., asking participants how much they drank post-stressor, rather than measuring it directly) or used only subjective stress ratings without cortisol data. However, the use of validated self-report questionnaires, such as the Alcohol Use Disorders Identification Test (AUDIT), was permissible when used for the initial diagnostic classification of participants’ AUD status. Studies involving non-human subjects, human subjects characterized by a mean age below 18 (e.g., studies of cortisol in infants after maternal alcohol consumption during pregnancy) or selected for other psychiatric disorders than AUD (e.g. studies exploring comorbidity between post-traumatic stress disorder and AUD) were also excluded (see Fig. 1B).

### Data extraction and preparation

Data extraction and preparation were conducted collaboratively, with LJW conducting the literature search and information extraction, while RM performed quality control and carried out the analysis. Proceeding from the 166 studies assessed for eligibility by full-text screening, study inclusion was validated with an inter-rater agreement of Cohen’s κ = 0.93 and a sensitivity of 96%. Any discrepancies regarding study inclusion were resolved through thoughtful discussion. Purely graphical data was extracted manually using the WebPlot Digitizer [39]. No queries for unreported data were submitted to corresponding authors of those 82 studies that did not meet the inclusion criteria. All extracted information and analysis code is provided online: https://osf.io/m743u/. The key information used in the analyses encompassed:

1. **Bibliography**: First author, title, year of publication.
2. **Baseline characteristics**: Sample size, mean age, percent male subjects, country of study, psychiatric diagnosis (method: questionnaire, medical examination, clinical interview; comorbidities allowed: yes, no, some);
3. **Sample characteristics**: AUD status and FH status (see below for more detailed information), and where appropriate the mean amount of pure alcohol consumed (in gram per kilogram g body weight, referenced to 70 kg) as proxy of an alcohol dose;
4. **Intervention characteristics**: Alcohol type (wine, beer, alcohol-free, favourite, long-drink, intravenous), stressor type: guided imagery emotion-induction, social (e.g., ego-threatening), cognitive (e.g., task or problems), physiologic (e.g., surgery, sauna), pharmacologic (cortisol-effective drugs).
5. **Outcome characteristics**: Mean and standard deviation of consumed amount of alcohol in millilitres (A_Vol_), mass of consumed pure alcohol in grams (A_Mass_), minimum cortisol levels (C_Min_), the difference between maximum and minimum cortisol levels (C_MaxMin_), and area under the cortisol concentration-time curves (C_AUC_).

#### AUD status

In line with modern diagnostic standards (e.g. DSM-5), empirical findings that challenge the separability of alcohol “abuse”/“misuse” and “dependence” (particularly with regard to disorder severity) and a lacking rationale to distinguish these disorders with respect to cortisol [26, 40], we opted for a dimensional AUD concept [41, 42]. However, most included studies relied on now deprecated diagnostic frameworks and according stigmatizing language. Hence, the AUD status of the respectively investigated individuals was coded to indicate broad categories of the spectrum of alcohol consumption behaviour that is hypothesized to induce adaptations in the cortisol stress processing system [26, 27]. AUD status was categorized as “AUD+” when actively drinking individuals were investigated, who were labelled as “alcoholics” (or patients suffering from alcohol “dependence” or AUD) in the respective study *and* who were diagnostically assessed using disorder-specific instrument(s). Individuals who also met those criteria but were currently abstinent from drinking were categorized as “AUD±”. Individuals who were neither labelled as “alcoholics” nor diagnostically excluded for this reason (implying a mixture of individuals displaying no drinking, social drinking, and intentionally disguised problematic drinking) were categorized as “AUD-“. To address the according discrepancy between the “AUD-“ status and being healthy when according diagnostic information were not reported, we additionally coded if participants with comorbid disorders were included (cf. Figure 1C). The diagnostic instruments used by the studies were clinical interviews, medical examinations, and alcohol-specific questionnaires, including the AUDIT, Missouri Alcohol Severity Scale (MASS) and Short Michigan Alcohol Screening Test (MAST) [43–45] (see data on https://osf.io/m743u for details). The specific instrument was coded to evaluate the robustness of the estimated effects to between-study heterogeneity.

#### FH status

Similar to AUD status, FH status was categorized as “FH+” when the investigated individuals explicitly reported a positive family history of AUD and were termed accordingly by the investigators. Individuals who explicitly reported no family history of AUD were categorized as “FH-“, whereas individuals who were not assessed in this regard were categorized as “FH±”. To enable the informed imputation of FH± individuals, we extracted and coded the lifetime prevalence rates of AUD, including alcohol “abuse” and “dependence”, for the respective country or cultural area in which each of the concerned studies were conducted [41, 46, 47].

Risk of bias of each study was assessed using the Cochrane Collaboration Risk of Bias (2.0) tool for non-randomized (intervention) studies [48]. This scoring includes the assessment of various dimensions including bias related to subject selection/assignment, deviations from intended intervention/misclassification of allocated interventions, measurement of outcomes, missing data, selection of results or repetitive publication, and neglect of confounding variables.

### Statistical analysis

Our primary analysis relied on three main sets of multilevel meta-regression models, each corresponding to a key research question: (A) the effect of acute stress on alcohol consumption, (B) the effect of acute alcohol consumption on cortisol responses without a concurrent stressor, and (C) the moderating role of concurrent stress on alcohol-related cortisol responses. To this end, we analysed the following effect sizes [49] from 40 included experimental studies (cf. Figure 1A).

1. **Stress-related alcohol consumption:** The standardized mean difference in alcohol consumption between acute stress compared to control conditions, quantified by the volume of alcohol consumed in millilitres (A_Vol_) and the corresponding mass of pure alcohol in grams (A_Mass_).
2. **Alcohol-related cortisol response**: The standardized mean difference in various non-compartmental cortisol measures following alcohol versus placebo consumption, encompassing differences between maximum and minimum cortisol levels (C_MaxMin_), minimum cortisol levels (C_Min_) and cortisol concentration-time curves (C_AUC_). Related to pharmacokinetic concepts, C_MaxMin_ indicates the magnitude by which cortisol levels increase in response to stressor exposure or alcohol consumption (reflecting cortisol *reactivity*), while C_Min_ is reflecting the fractional turnover of cortisol, that is, the decline of cortisol levels towards a steady state concentration (reflecting cortisol *recovery*) [23]. Higher levels of C_Min_ indicate a slower convergence towards higher steady state concentrations and thus a poorer regulatory capacity to re-regulate stress or alcohol-induced increases in cortisol levels.
  Only 13 studies linking (1) acute alcohol consumption and (2) alcohol-related cortisol responses in AUD± individuals used interventions, whereas the majority of studies on cortisol responses in individuals with various AUD statuses (incl. AUD±) were purely observational (31 out of 44; cf. Figure 1A). Those studies reported cortisol levels after various stressor or drug exposure procedures but lacked appropriately randomized control conditions, hindering the extraction unbiased effect sizes to infer the causal effect of stress or drugs on cortisol. Therefore, we also relied on the meta-analytic pooling of the following effect sizes despite of their sensitivity to confounding and collider bias:
3. **Observed cortisol response**: The baseline-standardized difference between maximum and minimum cortisol levels (C_MaxMin_; assuming a correlation between C_Max_ and C_Min_ of *r* = 0.75) and C_Min_ within different arms of observational and interventional studies as a function of AUD status.

Given the considerable variability in the study-level characteristics extracted from all included studies, we conducted random-effects meta-analyses using the *metafor* package [50] in R, version 3.4.2 statistical software [51]. Most studies reported various alcohol consumption or cortisol-related outcomes, yielding correlated effect sizes that depended on shared study-level characteristics. To account for this dependency, we fitted separate multilevel meta-regression models to each outcome assuming conditional within-study homogeneity and between-study heterogeneity of effect sizes [52]. Noteworthy, the results hardly changed when the dependency between different outcomes was explicitly modelled (see supplementary material).

Proceeding from this approach, we explored the sensitivity of the random-effects estimates (REE) to various study-level moderators (AUD status; FH status; Stressor type: no stressor/control vs. imagery vs. social-evaluative vs. physiologic vs. cognitive vs. pharmacologic/CRH) and small-study effects, including potential publication bias. The latter was checked by contour-enhanced funnel plots [53] and adjusted for by obtaining a precision-effect estimate with standard error (PEESE) [54]. While acknowledging that the PEESE may slightly underestimate the true association in the presence of questionable research practices, simulations suggest that it provides the most precise estimates when residual effect heterogeneity and small-study effects are present [55]. Due to the large number of studies exclusively investigating FH± individuals, we treated FH status as a metric predictor ranging from 0% (FH- individuals) to 100% (FH+ individuals), where FH± was imputed using the respective AUD lifetime prevalence (see “Data extraction and preparation”). The sensitivity of that reference imputation to systematic over- or underestimation, was investigated through alternative imputations using AUD lifetime prevalence multiplied by a large set of scaling factors (effectively covering FH status scenarios ranging from 1% to 97%). Finally, the sensitivity to exclusion of FH± individuals was checked. All reported results are robust to those specifications (see supplementary material).

## Results

Our systematic review identified 84 primary studies, providing 386 effect sizes from 3919 individuals (79.2% male), that quantitatively examined the relationship between acute stress, alcohol consumption, and cortisol levels. The following sections report the findings from the meta-analyses of the 40 included experimental studies. Approximately 85% of the information contributed by those studies was characterized by “low risk of bias” or “some concerns” (cf. supplementary Figures S3 and S4). The exploratory meta-analysis of the remaining 44 studies providing observational data is reported in the supplementary material. Please note that the descriptive language used in this section refers to the most likely effect direction, but does not imply statistical significance unless this is explicitly stated.

### Meta-analysis of stress-related alcohol consumption

**Fig. 2:**
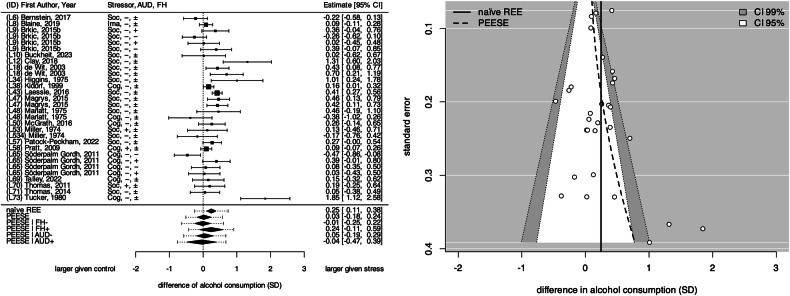
Forest and funnel plot of the reported and estimated effects of acute stress exposure on alcohol consumption behaviour (A_Vol_). (**Left**) Forest plot. The dotted vertical line signifies the scenario of “no stress-related alcohol consumption”. Square symbols indicate the effect size reported by the respective sample, with their size indicating relative number of individuals informing that sample. Error bars indicate 95% confidence interval (CI) around each estimate. Diamond symbols indicate meta-analytical effect estimates. REE: naïve random-effects estimate, PEESE: precision effect estimate with standard error, SD: standard deviations. (**Right**) Contour-enhanced funnel plot highlighting several small study effects. AUD+= actively drinking individuals meeting AUD criteria, AUD-= individuals with no AUD diagnosis; FH±= no reported family history of AUD, FH+=positive FH of AUD, FH-=negative FH of AUD; Stressor type: cognitive, imagery, social-evaluative. A complete reference list of the included studies is provided as  supplementary material.

To examine alcohol consumption after an acute stress compared to control procedure, we analysed n = 20 experimental studies, contributing a total of k = 52 effect sizes (A_Vol_: 30, A_Mass_: 22), and encompassed 1329 individuals with a mean age 18.7 to 46.6 years. 50.83% were male and 49.17% female, 12.11% were AUD+ individuals, whereas 87.89% were AUD- individuals. 5.12% had a positive or negative family history of AUD (FH+/FH-), whereas for 94.88% no family history of AUD was reported (FH±). Figure 2A presents the standardized mean differences in alcohol consumption A_Vol_, along with various meta-analytically derived effect estimates. The naïve REE suggested that stressor exposure significantly increases alcohol consumption (A_Vol_: g_REE_ = 0.25 SD, SE = 0.07; CI_95%_= [0.11, 0.39] and A_Mass_: g_REE_ = 0.28 SD, SE = 0.08; CI_95%_= [0.13, 0.44]). However, Fig. 2B reveals that smaller studies, which reported larger effects, disproportionately influenced this estimate, often approaching the significance threshold in favour of increased consumption after stress. Adjusting for these *small study effects* effectively attenuated both effect estimates (A_Vol_: g_PEESE_ = 0.02 SD, SE = 0.11; CI_95%_= [−0.19, 0.23] and A_Mass_: g_PEESE_ = 0.07 SD, SE = 0.12; CI_95%_= [−0.15, 0.30]).

#### Meta-regression of stress-related alcohol consumption on sample-level covariates

The residual heterogeneity in alcohol consumption (A_Vol_) was significant (τ = 0.27; Q = 83.42; p < 0.001), indicating the presence of potential moderators such as differences in baseline, sample, intervention or outcome characteristics across the included studies. To explore these moderators, we performed several meta-regression analyses summarized in Table 1.

**Table 1:** Meta-regression of stress-related alcohol consumption A_Vol_ on potential study-level moderators.

		Volume of consumed alcohol in ml, A_Vol_			
Regressor	k	Estimate (SE)	[95% CI]	Q_M_(df)	p value
Baseline					
Age in years (y)	27			0.87 (1)	0.35
Ø 18.7 y		+0.15 SD (0.11)	[−0.06, 0.35]		
Ø 24.5 y		+0.10 SD (0.09)	[−0.07, 0.27]		
Ø 46.6 y		−0.08 SD (0.20)	[−0.47, 0.32]		
Percent male subjects	30			0.55 (1)	0.46
Ø 0%		−0.11 SD (0.20)	[−0.50, 0.29]		
Ø 50%		+0.03 SD (0.11)	[−0.19, 0.25]		
Ø 100%		+0.17 SD (0.23)	[−0.28, 0.62]		
Assessment of AUD	25			6.75 (1)	0.01
Interview		−0.03 SD (0.09)	[−0.21, 0.16]		
Questionnaire		+0.30 SD (0.12)	[0.06, 0.53]		
Comorbidity inclusion	18			0.15 (1)	0.70
No		+0.04 SD (0.12)	[−0.19, 0.26]		
Some		+0.10 SD (0.17)	[−0.22, 0.43]		
Sample					
AUD status	30			0.02 (1)	0.89
AUD-		+0.02 SD (0.12)	[−0.21, 0.25]		
AUD+		−0.01 SD (0.20)	[−0.40, 0.39]		
FH status	30			2.86 (1)	0.09
Ø 0% FH+ (=FH-)		−0.02 SD (0.12)	[−0.24, 0.21]		
Ø 10% FH+		+0.02 SD (0.12)	[−0.21, 0.24]		
Ø 100% FH+		+0.23 SD (0.17)	[−0.11, 0.57]		
Dose consumed per kg bodyweight	30			0.01 (1)	0.92
Ø 0 g		+0.03 SD (0.12)	[−0.21, 0.26]		
Ø 0.2 g		+0.02 SD (0.11)	[−0.19, 0.24]		
Ø 0.6 g		+ 0.01 SD (0.16)	[−0.30, 0.32]		
Intervention					
Alcohol type	30			1.53 (4)	0.82
Favourite		+0.12 SD (0.32)	[−0.51, 0.75]		
Alcohol-free		−0.07 SD (0.19)	[−0.45, 0.31]		
Wine		−0.49 SD (0.43)	[−1.33, 0.36]		
Long drink		−0.05 SD (0.18)	[−0.40, 0.29]		
Beer		−0.05 SD (0.16)	[−0.36, 0.25]		
Stressor type	30			0.18 (2)	0.91
Social evaluative		+0.03 SD (0.15)	[−0.25, 0.32]		
Imagery		+0.03 SD (0.32)	[−0.59, 0.65]		
Cognitive		−0.04 SD (0.16)	[−0.35, 0.27]		

All estimates are adjusted for the precision of effect estimates (PEESE). Moderator levels that could not be estimated due to unavailable data (e.g., data on alcohol-related cortisol reactivity or recovery in currently abstinent individuals who previously met AUD criteria AUD±) are not reported. When moderators lacked discrete levels, their minimum, median and maximum values were contrasted.

*k* number of study samples, *AUD* alcohol use disorder.

Notably, the stress-related increase in A_Vol_ was more pronounced in FH+ individuals compared to FH- individuals (β = 0.25 SD, SE = 0.15; CI_95%_= [−0.04, 0.54]; see also Fig. 2A). This moderating effect was even stronger for A_Mass_ (β = 0.60 SD, SE = 0.19; CI_95%_= [0.22, 0.98]). In contrast, AUD status did not appear to moderate the effect of stressor exposure on alcohol consumption, possibly due to the limited number of studies assessing AUD+ individuals (4 out of 20 studies) [56–59]. Regarding further potential moderators, we observed that particularly studies assessing AUD status through questionnaires rather than clinical interviews reported increased alcohol consumption after stress.

### Meta-analysis of alcohol-related cortisol response

To examine cortisol responses after alcohol compared to placebo consumption, without any concurrent stressor exposure, we analysed n = 15 experimental studies, providing a total of k = 63 effect sizes (C_MaxMin_: 30, C_Min_: 26, C_AUC_: 7), and encompassed 426 individuals with a mean age 20.0 to 33.5 years. 72.23% were male and 27.77% female, 6.57% were AUD+ individuals, whereas 93.43% were AUD- individuals. 52.58% had a positive or negative family history of AUD (FH + /FH-), whereas for 47.42% no family history of AUD was reported (FH±).

**Fig. 3:**
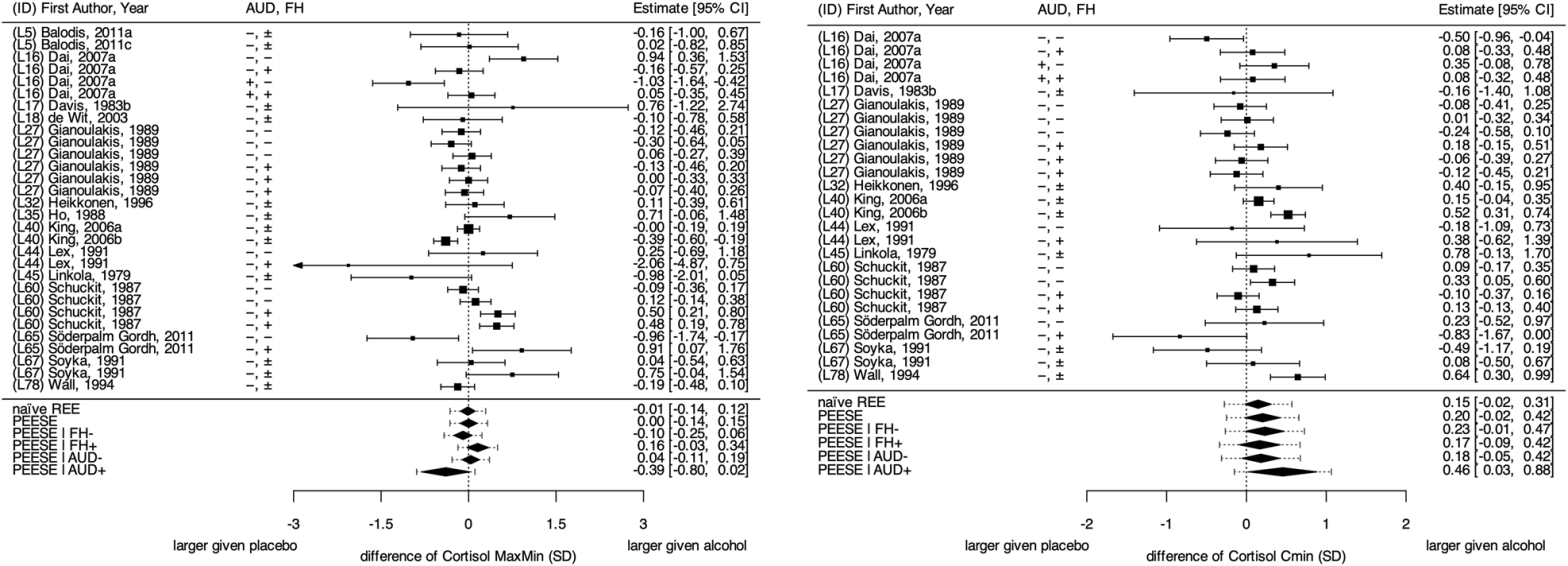
Forest plots of the reported and estimated effects of acute alcohol exposure on cortisol. Cortisol is indicated by (**Left**) differences between the maximum and minimum cortisol levels (C_MaxMin_) and (**Right**) the minimum cortisol levels (C_Min_). The dotted vertical line signifies the scenario of “no efficacy of acute alcohol consumption”. Square symbols indicate the effect size reported by the respective sample, with their size indicating relative number of individuals informing that sample. Error bars indicate 95% confidence interval (CI) around each estimate. Diamond symbols indicate meta-analytical effect estimates. REE: naïve random-effects estimate, PEESE: precision effect estimate with standard error. AUD + = actively drinking individuals meeting AUD criteria, AUD- = individuals with no AUD diagnosis; FH± = no reported family history of AUD, FH + =positive FH of AUD, FH- =negative FH of AUD. A complete reference list of the included studies is provided as supplementary material.

Figure 3 presents the standardized mean differences in C_MaxMin_ (*reactivity*) and C_Min_ (*recovery*), along with various meta-analytically derived effect estimates. In the absence of small-study effects, acute alcohol consumption did not significantly predict C_MaxMin_ (g_REE_ = −0.01 SD, SE = 0.07; CI_95%_= [−0.14, 0.12] and g_PEESE_ = 0.00 SD, SE = 0.08; CI_95%_= [−0.14, 0.15]) indicating no substantial change in cortisol *reactivity*. By contrast, effects on C_Min_ suggested a slight decrease of alcohol-related cortisol *recovery* (C_Min_↑; g_REE_= 0.15 SD, SE = 0.08; CI_95%_= [−0.02, 0.31] and g_PEESE_ = 0.20 SD, SE = 0.11; CI_95%_= [−0.02, 0.42]). Similarly, the analysis of C_AUC_ showed a consistent increase after acute alcohol as compared to placebo consumption (g_REE_= 0.38 SD, SE = 0.28; CI_95%_= [−0.16, 0.93]) unless *small study* effects in this analysis set were accounted for (g_PEESE_ = −0.18 SD, SE = 0.15; CI_95%_= [−0.47, 0.11], see supplementary Figure S1).

#### Meta-regression of alcohol-related cortisol responses on sample-level covariates

The residual heterogeneity of alcohol-related cortisol *reactivity* (C_MaxMin_; τ = 0.15; Q = 87.38; p< 0.001) and *recovery* (C_Min_; τ = 0.20; Q = 55.34; p< 0.001) across this analysis set was significant (C_AUC_; τ = 0.00; Q = 8.49; p = 0.13), indicating the presence of potential moderators. To explore these moderators, we performed several meta-regression analyses summarized in Table 2.

**Table 2:** Meta-regression of alcohol-related cortisol *reactivity* without concurrent acute stress exposure (C_MaxMin_, difference between maximum and minimum levels) and *recovery* (C_Min_, minimum levels) on potential study-level moderators.

	Cortisol reactivity, C_MaxMin_					Cortisol recovery, C_Min_				
Stratum	k	Estimate (SE)	[95% CI]	Q _M_(df)	p value	k	Estimate (SE)	[95% CI]	Q _M_(df)	p value
**Age in years**	27			0.36(1)	0.55	24			0.80 (1)	0.37
Ø 20.0 yrs		+0.07 (0.13)	[−0.18, 0.32]				+0.37 (0.19)	[0.00, 0.74]		
Ø 25.3 yrs		+0.01 (0.08)	[−0.14, 0.16]				+0.25 (0.12)	[0.01, 0.49]		
Ø 33.5 yrs		−0.08 (0.17)	[−0.41, 0.25]				+0.07 (0.22)	[−0.36, 0.51]		
**Percent male subjects**	30			3.13 (1)	0.08	26			0.25 (1)	0.62
Ø 0%		−0.27 (0.16)	[−0.59, 0.05]				+0.08 (0.28)	[−0.46, 0.63]		
Ø 100%		+0.08 (0.08)	[−0.07, 0.24]				+0.24 (0.14)	[−0.03, 0.51]		
**Assessment of AUD**	22			1.57(1)	0.21	21			0.91 (1)	0.34
Interview		−0.08 (0.08)	[−0.24, 0.09]				+0.13 (0.16)	[−0.19, 0.45]		
Questionnaire		+0.09 (0.11)	[−0.12, 0.31]				+0.35 (0.19)	[−0.01, 0.72]		
**Comorbidity inclusion**	23			0.01(1)	0.91	22			0.55 (1)	0.46
No		−0.06 (0.26)	[−0.57, 0.46]				+0.21 (0.12)	[−0.02, 0.44]		
Some		−0.03 (0.08)	[−0.18, 0.13]				−0.08 (0.41)	[−0.89, 0.72]		
Sample										
**AUD status**	30			3.21(1)	0.07	26			1.93 (1)	0.16
AUD-		+0.03 (0.08)	[−0.13, 0.19]				+0.18 (0.13)	[−0.06, 0.43]		
AUD+		−0.35 (0.21)	[−0.77, 0.07]				+0.46 (0.22)	[0.03, 0.90]		
**FH status**	30			7.17(1)	0.01	26			0.63 (1)	0.43
Ø 0% FH+ (=FH-)		−0.08 (0.08)	[−0.23, 0.08]				+0.23 (0.11)	[0.01, 0.44]		
Ø 10% FH+		−0.05 (0.07)	[−0.19, 0.10]				+0.22 (0.11)	[0.00, 0.43]		
Ø 100% FH+		+0.16 (0.09)	[−0.03, 0.34]				+0.16 (0.12)	[−0.08, 0.39]		
**Dose consumed per kg Bodyweight**	30			0.30(1)	0.59	26			3.38 (1)	0.07
Ø 0.1 g		+0.04 (0.11)	[−0.16, 0.25]				+0.08 (0.13)	[−0.18, 0.34]		
Ø 0.5 g		+0.01 (0.08)	[−0.14, 0.16]				+0.21 (0.11)	[−0.02, 0.43]		
Ø 2.0 g		−0.13 (0.26)	[−0.65, 0.38]				+0.69 (0.29)	[0.13, 1.25]		
Intervention										
**Alcohol type**	29			1.87(1)	0.17	25			0.36 (1)	0.55
Iv		+0.05 (0.08)	[−0.10, 0.20]				+0.18 (0.13)	[0.07, 0.44]		
Long drink		−0.18 (0.15)	[−0.47, 0.11]				+0.33 (0.22)	[−0.09, 0.76]		

All estimates are adjusted for the precision of effect estimates (PEESE). Moderator levels that could not be estimated due to unavailable data (e.g., data on alcohol-related cortisol *reactivity* or *recovery* in currently abstinent individuals who previously met AUD criteria AUD±) are not reported. When moderators lacked discrete levels, their minimum, median and maximum values were contrasted.

*k* number of study samples, *iv* intravenous, *AUD* alcohol use disorder.

Most remarkably, FH+ individuals showed an increased alcohol-related cortisol *reactivity* compared to FH- individuals (C_MaxMin_↑; β = 0.23 SD, SE = 0.09; CI_95%_= [0.06, 0.41]; see also Fig. 3A). In contrast, AUD+ individuals showed a decreased cortisol *reactivity* compared to AUD- individuals (C_MaxMin_↓; β = −0.38 SD, SE = 0.21; CI_95%_= [−0.79, 0.04]). AUD+ individuals showed a decrease in alcohol-related cortisol *recovery* compared AUD- individuals (C_Min_↑; β = 0.28 SD, SE = 0.20; CI_95%_= [−0.11, 0.67]). Similarly, this alcohol-related decrease in cortisol *recovery* became more pronounced with the consumption of the highest doses of pure alcohol (C_Min_↑; β = 0.33 SD per g/kg bodyweight, SE = 0.18; CI_95%_= [−0.02, 0.68]), suggesting that the impact of acute alcohol consumption on cortisol *recovery* is dose-dependent.

### Meta-regression of alcohol-related cortisol response on additional acute stressor exposure

To investigate the moderation of alcohol-related cortisol responses by additional stressor exposure, we analysed the 15 studies without any stressor exposure (cf. “Meta-analysis of alcohol-related cortisol response”) and 9 further studies, that were characterized by concurrent alcohol and stressor exposure. In sum, this analysis included n = 24 experimental studies, provided a total of k = 115 effect sizes (contrasting alcohol vs. placebo consumption after stress with alcohol vs. placebo consumption without any stress or control condition; C_MaxMin_: 53, C_Min_: 46, C_AUC_: 16), and encompassed 983 individuals with a mean age 20.0 to 54.6 years. 72.54% were male and 27.46% female, 8.55% were AUD+ individuals, whereas 91.45% were AUD- individuals. 50.77% had a positive or negative family history of AUD (FH + /FH-), whereas for 49.24% no family history of AUD was reported (FH±).

Compared to alcohol consumption without any stressor exposure, alcohol consumption with stressor exposure resulted in significantly decreased cortisol *reactivity* (C_MaxMin_↓_;_ β = −0.25 SD, SE = 0.10; CI_95%_= [−0.45, −0.06]), significantly increased cortisol *recovery* (C_Min_↓; β = −0.21 SD, SE = 0.09; CI_95%_= [−0.40, −0.03]), and consistently decreased C_AUC_ (β = −0.52 SD, SE = 0.14; CI_95%_= [−0.80, −0.25]).

As presented in Table 3, alcohol-related decreases of cortisol *reactivity* (C_MaxMin_↓) was more pronounced if a pharmacological CRH-challenge or physiological stress-induction protocol was used ($\hat{{\rm{g}}}$_CRH_ = −0.63 SD, SE = 0.41, $\hat{{\rm{g}}}$_Physiologic_ = −0.43 SD, SE = 0.22) as compared to a cognitive or social-evaluative one ($\hat{{\rm{g}}}$_Cognitive_ = −0.24 SD, SE = 0.14, $\hat{{\rm{g}}}$_Social_ = −0.20 SD, SE = 0.14). The reverse effect pattern was observed with regard to cortisol *recovery*, suggesting a selectively increased alcohol-related cortisol *recovery* (C_Min_↓) when individuals were exposed to cognitive or social-evaluative stress-induction protocols ($\hat{{\rm{g}}}$_Cognitive_ = −0.35, $\hat{{\rm{g}}}$_Social_ = −0.20). With regard to C_AUC_, CRH challenge yielded the most pronounced alcohol-related decrease ($\hat{{\rm{g}}}$_CRH_ = −0.88 SD, SE = 0.38).

**Table 3:** Meta-regression of alcohol-related cortisol *reactivity* under concurrent acute stress exposure (C_MaxMin_, difference between maximum and minimum levels) and *recovery* (C_Min_, minimum levels) on potential study-level moderators.

	Cortisol reactivity, C_MaxMin_					Cortisol recovery, C_Min_				
Stratum	k	Estimate (SE)	[95% CI]	Q _M_(df)	p value	k	Estimate (SE)	[95% CI]	Q _M_(df)	p value
**Age in years**	47			1.96(1)	0.16	41			0.03 (1)	0.86
Ø 20.0 yrs		+0.08 (0.08)	[−0.08, 0.24]				+0.11 (0.11)	[−0.16, 0.26]		
Ø 25.3 yrs		+0.01 (0.07)	[−0.12, 0.14]				+0.04 (0.09)	[−0.14, 0.23]		
Ø 33.5 yrs		−0.40 (0.30)	[−0.99, 0.19]				−0.02 (0.35)	[−0.71, 0.67]		
**Percent male subjects**	51			0.17 (1)	0.68	44			0.75 (1)	0.39
Ø 0%		+0.07 (0.19)	[−0.29, 0.43]				−0.12 (0.22)	[−0.55, 0.30]		
Ø 100%		−0.02 (0.09)	[−0.20, 0.16]				+0.10 (0.11)	[−0.12, 0.32]		
**Assessment of AUD**	38			15.14(1)	0.00	36			0.76 (1)	0.68
Interview		−0.12 (0.05)	[−0.21, −0.03]				+0.02 (0.12)	[−0.22, 0.25]		
Medical examination	−0.62 (0.32)	[−1.26, 0.02]				+0.06 (0.41)	[−0.76, 0.87]			
Questionnaire	+0.15 (0.06)	[0.02, 0.27]				+0.17 (0.14)	[−0.11, 0.45]			
**Comorbidity inclusion**	38			0.59(1)	0.44	36			0.01 (1)	0.94
No		−0.15 (0.13)	[−0.41, 0.12]				+0.19 (0.19)	[−0.31, 0.43]		
Some		−0.03 (0.07)	[−0.18, 0.11]				+0.08 (0.09)	[−0.11, 0.26]		
Sample										
**AUD status**	53			2.19(1)	0.14	46			5.42 (1)	0.02
AUD-		+0.01 (0.07)	[−0.12, 0.14]				+0.03 (0.09)	[−0.16, 0.21]		
AUD+		−0.21 (0.16)	[−0.52, 0.09]				+0.35 (0.16)	[0.04, 0.66]		
**FH status**	53			5.79(1)	0.02	46			2.05 (1)	0.15
Ø 0% FH+ (==FH-)		−0.05 (0.07)	[−0.20, 0.09]				+0.07 (0.09)	[−0.10, 0.24]		
Ø 10% FH+		−0.04 (0.07)	[−0.19, 0.10]				+0.07 (0.09)	[−0.10, 0.24]		
Ø 100% FH+		+0.12 (0.09)	[−0.05, 0.29]				−0.02 (0.10)	[−0.22, 0.17]		
**Dose consumed per kg Bodyweight**	53			0.06(1)	0.80	46			1.40 (1)	0.24
Ø 0.1 g		−0.02 (0.09)	[−0.20, 0.16]				−0.03 (0.11)	[−0.26, 0.19]		
Ø 0.5 g		−0.01 (0.07)	[−0.15, 0.13]				+0.03 (0.10)	[−0.16, 0.21]		
Ø 2.0 g		+0.03 (0.49)	[−1.29, 0.61]				+0.24 (0.19)	[−0.13, 0.62]		
Intervention										
**Alcohol type**	50			5.13 (1)	0.02	43			1.13 (1)	0.29
Iv		+0.04 (0.07)	[−0.09, 0.18]				+0.03 (0.09)	[−0.15, 0.21]		
Long drink		−0.36 (0.17)	[−0.69, −0.03]				+0.23 (0.19)	[−0.14, 0.60]		
**Stressor type**	53			8.22(4)	0.08	46			9.62 (4)	0.05
No stress exposure		+0.13 (0.09)	[−0.06, 0.31]				+0.15 (0.09)	[−0.02, 0.32]		
Cognitive		−0.11 (0.15)	[−0.40, 0.18]				−0.20 (0.13)	[−0.46, 0.07]		
Pharmacological (CRH)		−0.50 (0.41)	[−1.31, 0.30]				+0.25 (0.39)	[−0.52, 1.02]		
Physiologic		−0.30 (0.21)	[−0.72, 0.12]				+0.21 (0.19)	[−0.17, 0.59]		
Social-evaluative		−0.07 (0.12)	[−0.30, 0.16]				−0.05 (0.11)	[−0.27, 0.16]		

All estimates are adjusted for the precision of effect estimates (PEESE). Moderator levels that could not be estimated due to unavailable data (e.g., data on alcohol-related cortisol *reactivity* or *recovery* in currently abstinent individuals who previously met AUD criteria AUD±) are not reported. When moderators lacked discrete levels, their minimum, median and maximum values were contrasted. Please note that Table 3 is based on the same set of studies as Table 2 and ten additional studies featuring concurrent acute stress exposure. Meta-regression of C_AUC_ are reported in the supplementary material.

*k* number of study samples, *iv* intravenous, *AUD* alcohol use disorder.

Importantly, these effect estimates are susceptible to confounding and collider (e.g. selection) biases, as they largely rely on studies that assessed alcohol-related cortisol measures either with or without a concurrent stressor exposure but did not randomly cross both, alcohol versus placebo and stress versus control treatments. However, sensitivity analyses using a subset of five studies with rigorous experimental controls (crossing both alcohol vs. placebo and stress vs. control conditions) showed consistent results, with decreased alcohol-related cortisol *reactivity* under stress as compared to control conditions (C_MaxMin_↓: β = −0.28 SD, SE = 0.14; CI_95%_= [−0.55, −0.01]) and a negligibly affected *recovery* (C_Min_: β = −0.18 SD, SE = 0.14; CI_95%_= [−0.46, 0.10]) [15, 21, 60–62].

## Discussion

Our systematic review and meta-analysis suggests that (1) acute stress does not increase acute alcohol consumption when *small study* effects are accounted for and (2) acute alcohol consumption seems to reduce cortisol *recovery* (particularly in AUD+ individuals, to a considerably lower extent in FH+ individuals, and when high doses around 2 g of pure alcohol per kg bodyweight are consumed). The analyses provided (3) no support of a substantial alcohol-induced increase in cortisol *reactivity* beyond placebo consumption. FH+ individuals show increased alcohol-related cortisol *reactivity*, whereas AUD+ individuals show decreased cortisol *reactivity*. Surprisingly, concurrent exposure to stressors modifies the absent alcohol effect on cortisol *reactivity* to (4) a reduced cortisol *reactivity* when alcohol is consumed under acute stress.

### The moderating impact of AUD and FH status on alcohol-related cortisol responses

AUD and FH status emerge as important predictors of alcohol consumption and variations in cortisol *reactivity* and *recovery* [4, 21, 63, 64]. However, when interpreting these findings, it is crucial to recognize that the AUD+ status relies on the diagnostic assessments of the included primary studies, which were methodologically diverse [65–67]. Therefore, while our analysis reveals distinct patterns associated with an AUD+ status, these should be understood as average trends within the available literature rather than universal effects of AUD. An AUD+ status is associated with markedly reduced alcohol-related cortisol *recovery* (C_Min_↑) and *reactivity* (C_MaxMin_↓), but is not associated with changes in the stress-induced increase in alcohol consumption. Conversely, FH+ individuals tentatively seem to show reduced cortisol *recovery* (C_Min_↑) and increased cortisol *reactivity (*C_MaxMin_↑), and an augmented stress-induced increase in acute consumption of alcoholic beverages.

Interestingly, the general AUD status (cf. Table 3) appears to have minimal impact on cortisol *reactivity* (i.e., the amount of secreted cortisol) when alcohol is consumed under concurrent stressor exposure or on stress-related alcohol consumption (A_Vol_, A_Mass_), which could affect the amount of secreted cortisol if consumed in high doses. Instead, its substantial effect on cortisol *recovery* under these conditions might be of a regulatory nature [23]. Since cortisol *recovery* reflects cortisol degradation towards a steady state level of cortisol (i.e., fractional turnover rates that effectively determines cortisol regulation) [23], a similarly reduced cortisol *recovery* (C_Min_↑) in AUD+ and AUD- individuals but a positive family history of AUD (FH+) may indicate a less effective cortisol regulation, characterized by slower cortisol degradation towards higher steady state levels after alcohol consumption under stress. This could imply a shared, possibly genetically driven predisposition towards less efficient cortisol regulation potentially exacerbating as AUD progresses [19, 29, 30].

On a speculative note, increased cortisol *reactivity* in individuals with a positive FH of AUD (FH+) could indicate an increased demand for alcohol-related cortisol-stress regulation, manifesting as increased stress-induced alcohol consumption. As AUD develops, this heightened cortisol *reactivity* may decrease, alongside a diminishing appeal of alcohol consumption to regulate cortisol-stress responses [26]. This could be reflected in a lack of substantial impact of general AUD status on acute alcohol consumption and a reduced cortisol *reactivity* in AUD+ individuals. While these hypotheses require further investigation, along with the presumed potential of C_Min_ as a biomarker to indicate risks of AUD development, our analyses underscores the importance of assessing the family histories of AUD in conjunction with AUD status when examining the relation between stressor exposure, alcohol consumption and cortisol responses [68].

### Dose-dependency of alcohol effects on cortisol responses

Our findings challenge the prevailing assumption that acute alcohol consumption inherently acts as a stressor, thereby increasing cortisol *reactivity* (C_MaxMin_↑) beyond what is observed after a placebo. This observation is specifically tied to the quantities of alcohol that was actually consumed in the studies we analysed, which ranged from 1.17 g to 2.0 g of pure alcohol per kilogram bodyweight, particularly noting those in a higher dose range exceeding 1 g of pure alcohol per kg bodyweight (that was met by 4 out of the 20 contributing studies) [69–73]. For a 70 kg individual, this equates to the consumption of 82 g to 140 g of pure alcohol, or roughly 2 to 3.5 litres of beer, 0.75 to 1.3 litres of wine, or 65 to 444 ml of 40 Volume % hard liquor. Our analyses indicate that increasing the quantity of consumed alcohol decreases cortisol *recovery* (C_Min_↑) but had virtually no effect on the cortisol *reactivity* (cf. Tables 2 and 3). Remarkably, this pattern of effects at high doses mirrors those observed in AUD+ individuals [21, 63, 74]. Therefore, our analyses suggest that higher doses of alcohol may indeed act as stressors, potentially increasing cortisol *reactivity*, although the relevance of such high doses exceeding 2.0 g of pure alcohol per kg/bodyweight to typical alcohol consumption patterns among individuals meeting AUD criteria remains debatable [32, 75]. The precise pharmacodynamics of an effective alcohol dose in terms of impacting cortisol *reactivity* cannot be formally explored in our meta-analyses. However, as long as the quantity of consumed alcohol remained below 1 g per kilogram bodyweight, doubling the quantity has virtually no effect on cortisol *recovery* [4, 63, 76]. It therefore seems reasonable to suggest that studies using high doses of alcohol should administer relative doses exceeding the maximum dose of 2.0 g per kg bodyweight threshold. Comparable to the dose restriction of our meta-analysis, the majority of studies that contributed to our analyses involved alcohol self-administration paradigms in relatively young and healthy individuals, as indicated by the maximum mean ages reported in Table 2 and Table 3, all below 40 years [38]. Thus, stress-induced changes in alcohol consumption and stress-/drug-induced cortisol responses in older individuals, particularly those beyond the mean age of AUD onset (mid-40s), or in abstinent individuals who previously met AUD criteria (irrespective of ethical considerations) might differ from our findings [35–38]. These differences could be due to age-related impairments or long-term effects of alcohol consumption [35, 77]. Consequently, our analyses highlight the need for future research to include higher doses of alcohol and older individuals with diverse statuses of AUD and FH of AUD, to enhance our understanding of alcohol-related changes in cortisol responses and to refine models of AUD development and progression [19, 32].

### Alcohol effects on cortisol responses under concurrent acute stressor exposure

**Fig. 4:**
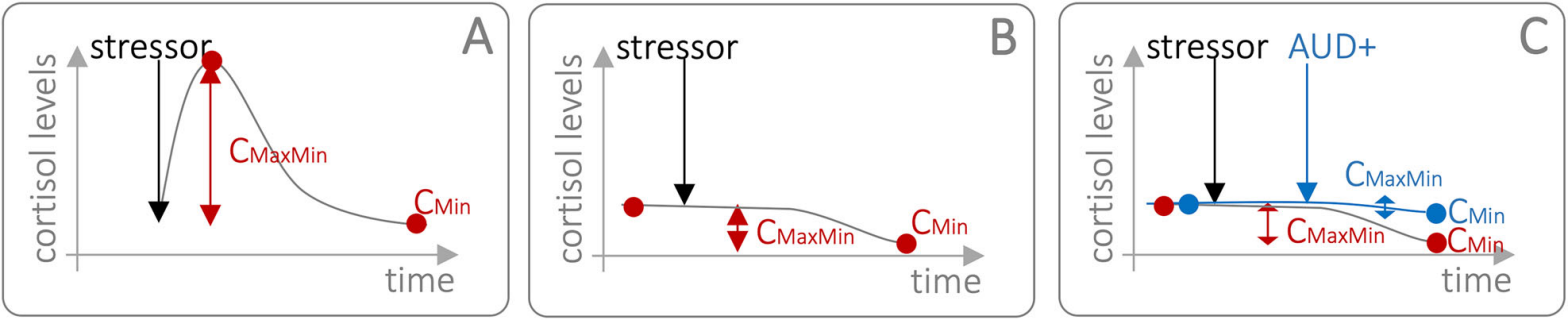
Cortisol Trajectories After Stressor Exposure. Schematic illustration of (**A**) a stress-induction protocol that successfully increases cortisol levels, with higher C_MaxMin_ indicating higher magnitudes of cortisol secretion in response to the stressor (*reactivity*). If (**B**) a stress-induction protocol does not induce any increase in cortisol levels, C_MaxMin_ effectively converges to C_Min_ (i.e., does no longer indicate the magnitude of a stressor-related cortisol response). If such an ineffective stress-induction protocol is implemented and actively abusing individuals with AUD (AUD+) show increased C_Min_ values and thus a reduced *recovery*, (**C**) might explain how the surprising moderation effect of additional acute stress exposure on cortisol *reactivity* (C_MaxMin_) might have been generated in the given set of studies.

Our analyses do not indicate a substantial effect of acute alcohol consumption on cortisol *reactivity* (C_MaxMin_) [31]. Surprisingly, when we included concurrent stressor exposure to explain some heterogeneity in this (missing) alcohol-related effect on cortisol *reactivity*, the effect of alcohol on cortisol reversed into decreasing *reactivity* and increasing *recovery* (C_MIn_↓). This occurs despite the absence of evidence suggesting the involvement of negative feedback loops that could diminish the efficacy of either stress or alcohol in elevating cortisol levels simultaneously [78]. As visualised in Fig. 4, we identified the calculation of cortisol *reactivity* (C_MaxMin_) along with the implementation of ineffective stress-induction protocols (with respect to increases of cortisol levels) as potential explanation for this unexpected effect pattern. In scenarios where stress-induction protocols fail to elevate cortisol levels (cf. Figure 4B), the natural circadian decline in cortisol levels could unfortunately also result in apparent increases in C_MaxMin_ values, even though there is no actual stress-induced cortisol secretion or increase (i.e., such C_MaxMin_ values would effectively reflect the same process(es) as C_Min_ values) [23]. Indeed, Fig. 4C demonstrates how increases in C_Min_ associated with an AUD+ status may further decrease C_MaxMin_, potentially explaining some of our more surprising findings. However, this rationale does not hold for similar patterns observed following CRH administration, which increases cortisol levels more clearly. Given that C_MaxMin_ reflects one of the most important concepts in relation to acute stress (i.e., stress *reactivity*), it is crucial for future studies to eliminate this confounding factor by implementing stress-induction protocols that effectively elevate cortisol levels, particularly in studies exploring the interaction between acute stress, alcohol consumption and cortisol levels in relation to AUD status. This approach will help clarify the dynamics of cortisol responses under these conditions.

### On the specificity of stress-related increases in alcohol consumption

Our systematic review with meta-analyses reveal that stressor exposure only increases acute alcohol consumption when *small study* effects are not controlled for. This finding raises concerns about potential publication biases and questions the specificity of the observed stress effect in relation to alcohol as opposed to non-alcoholic beverages [15, 79]. Although we did not explicitly analyse the consumption of non-alcoholic beverages, existing research offers mixed effects, with some studies supporting an alcohol-specific stress effect [61, 79, 80], while others present contrary evidence [15, 16, 81]. Additionally, some studies emphasize the impact of beliefs about alcohol effects (alcohol-related placebo effects) and the actual content of the beverage, rather than any pharmacologically specific effect of alcohol [62, 82]. Collectively, these findings alongside those from our analyses, suggest that the impact of acute stress on alcohol consumption may be less robust and more nuanced than traditionally posited by the SMH [3, 15, 19]. This implies a need for further research (including meta-analyses) to understand the conditions under which acute stress might affect alcohol consumption and to clarify the role of non-alcoholic alternatives in stress-related alcohol consumption.

### Research and translational implications

From a research perspective, our findings contribute to the discourse on the *CRH-dysregulation hypothesis*, an extension of the SMH [26, 83]. This hypothesis postulates that alcohol initially produces positive effects, which are subsequently countered by negative effects to restore homeostasis [4, 84]. These negative aftereffects, which intensify with repeated and/or increased alcohol consumption as AUD progresses, are thought to transition alcohol’s reinforcing properties from positive to negative [84]. The neuronal correlate of this transition is believed to be corticotropin-releasing hormone (CRH) and its (extra-)hypothalamic systems [26, 27, 84]. Since central CRH availability cannot be directly measured in humans, cortisol measures such as C_Min_ and C_MaxMin_ are used indicate this CRH-related latent process [19, 85].

Using these proxies, our systematic review with meta-analyses suggests that AUD+ individuals exhibit reduced cortisol *recovery* (C_Min_↑) and reduced cortisol *reactivity* (C_MaxMin_↓), especially when alcohol is consumed under concurrent stressor exposure. These findings support the idea that the CRH system synchronizes CRH and subsequent cortisol secretion in response to acute stress or alcohol consumption [19, 26, 86]. Frequent or intense re-exposure to stressors or alcohol is thought to decrease the sensitivity of the CRH system, potentially due to adaptations to excessive CRH and cortisol secretions, and may lead to increased basal levels of circulating cortisol, likely due to heightened intrinsic CRH activity and subsequent cortisol rhythmicity [28, 87].

From a clinical perspective, this reduced cortisol *reactivity* observed in AUD+ individuals may reflect an Addisonian-like stress intolerance (i.e., functional adrenal insufficiency) [20]. Signs of deficient adrenal responsiveness include hypotension, electrolyte imbalance, or weight loss [88]. Interestingly, this physiological blunting often coexists with heightened subjective negative affect during stressor exposure [89, 90], suggesting a dissociation between subjective and objective stress responses. Based on this reasoning, the assessment of C_Min_ (by a single, time-synchronized cortisol sample close to the diurnal nadir) alongside family history of AUD may help to identify patient of high risk of developing AUD early [91, 92]. Such patients could then be considered earlier for treatment in general, as well as for glucocorticoid pharmacotherapy [20, 93] and/or cognitive-behavioral therapy focused on stress reduction [91, 92, 94].

### Limitations

Several limitations should be considered when interpreting the findings of our meta-analysis. First, the majority of included studies recruited relatively young and healthy samples, with mean participant ages below 40 years. This limits the generalizability of our findings, particularly to older populations who have long-standing or remitted AUD given the known age-related physiological changes of the HPA axis and alcohol metabolism [35–38].

Second, most studies implemented moderate alcohol dosing protocols (typically ≤1.0 g/kg bodyweight), limiting our ability to reliably assess the dose-dependency of alcohol effects on cortisol *reactivity* and *recovery* [4, 63, 76]. Although exploratory analyses suggest that higher doses (>1.5 g/kg) may lead to more pronounced cortisol dysregulation, this finding is informed by only 5 effect estimates and may only generalize to individuals with severe AUD and a corresponding alcohol tolerance [95].

Third, between-study variability in stress-induction procedures and cortisol measurement protocols across studies has likely contributed to the effect heterogeneity we report. This includes the use of different biospecimens (e.g., saliva, serum, plasma) and biochemical assays. In particular, ineffective stress paradigms (that fail to reliably elevate cortisol levels) may confound interpretations of effects on cortisol reactivity (C_MaxMin;_ Fig. 4). Such variability also complicates efforts to assess the interactive effects of acute stress and alcohol consumption on HPA axis functioning, highlighting the need for validated stress-induction protocols and sampling schedules that reliably capture the full cortisol trajectories.

Fourth, our systematic review deliberately focused on cortisol as an objective biomarker of the physiological stress response [23]. Cortisol secretion is only one of many possible physiological mediators of psychological stress effects. We did not investigate subjective stress ratings as an outcome, which involves heterogeneous assessment instruments subjected to jingle-jangle fallacies [96]. The interplay between subjective psychological states and objective physiological responses remains a complex topic and has received its own separate investigation [18, 97].

Fifth, we highlighted that findings on stress-related increases in alcohol consumption may be affected by publication bias, as suggested by small-study effects. When controlling for those small-study effects, the previously reported stress-induced increase in alcohol consumption largely disappears, suggesting that the effect is subjected to confounding by investigator and participant expectations (incl. nocebo effects). Additionally, the very rare comparisons with non-alcoholic beverage consumption limits our ability to confirm the specificity of stress-induced alcohol intake.

Sixth, while we examined the moderating roles of AUD and FH status, the operational definitions of these categories varied across studies, and comorbid psychiatric or substance use disorders were not consistently diagnosed or reported. Hence, phenotyping was dependent on the diagnostic labels and assessment tools used in the primary studies [65, 66]. Crucially, specific data on the amount and frequency of alcohol consumption or the duration of abstinence for the AUD+ and AUD± groups, respectively, were often missing, precluding a more granular analysis of how AUD severity might impact the results. While this imprecise phenotyping is a significant constraint that likely contributed to effect heterogeneity within the different AUD groups, it also highlights the need for more detailed and standardized reporting in future primary research.

Finally, our meta-analyses are limited by the inherent constraints of aggregated data. We were unable to assess within-participant variability or individual differences in stress responsivity. Individual participant data meta-analyses is better suited to capture such nuanced effects in future work.

### Conclusion

Our systematic review with meta-analysis provides substantial support for the *CRH-dysregulation hypothesis* by showing that AUD+ status is linked with reduced cortisol *recovery* and *reactivity*, particularly under conditions of concurrent alcohol and acute stressor exposure. Conversely, our findings do not support the notion that alcohol induces excessive cortisol, and thus potentially CRH secretion, in individuals at risk for developing AUD (i.e., those with a positive family history of AUD) or in those without an AUD diagnosis (AUD-), regardless of additional stressor exposure. This might also be attributed to the alcohol dosing regimens implemented in the studies. Hence, our analyses reinforce the concept of cortisol (and potentially CRH) dysregulation in AUD but do not confirm the proposed mechanisms of its pathogenesis.

## Supplementary information

Supplementary Material

## Supplementary information

The online version contains supplementary material available at 10.17605/OSF.IO/M743U and 10.1038/s41398-025-03641-8.
