# Supplementary material for: A systematic review with meta-analysis on the relation between acute stress, alcohol consumption and cortisol levels in individuals with a personal, familial or no alcohol use disorder
Source: Transl Psychiatry. 2025 Oct 20;15:423. doi: 10.1038/s41398-025-03641-8 (PMC12537857; doi:10.1038/s41398-025-03641-8)
Supplement: Supplementary file 1 — Supplementary Material [file 41398_2025_3641_MOESM1_ESM.docx]

# Supplementary Analyses

## Meta-analysis of stress-related alcohol consumption

## Based on multivariate meta-regression modelling, stressor exposure significantly increases alcohol consumption (A_Vol_: g_REE_= 0.29 SD, SE= 0.10; CI_95%_= [0.10, 0.47] and A_Mass_: g_REE_= 0.28 SD, SE= 0.10; CI_95%_= [0.10, 0.47]). Adjusting for small study effects effectively attenuated both effect estimates (A_Vol_: g_PEESE_= -0.05 SD, SE= 0.13; CI_95%_= [-0.30, 0.21] and A_Mass_: g_PEESE_= -0.04 SD, SE= 0.13; CI_95%_= [-0.30, 0.21]). The residual heterogeneity in A_Vol_ (τ= 0.34) and A_Mass_ (τ= 0.36) was significant (Q= 154.32; p< .001). Both outcomes were collinear.

# Meta-regression of stress-related alcohol consumption on family history of AUD

## The stress-related increase in A_Vol_ was more pronounced in FH+ individuals compared to FH- individuals when samples with unknown FH status were not imputed but removed from the analysis set (β= 0.29 SD, SE= 0.16; CI_95%_= [-0.03, 0.61]). As shown below, this moderation effect was also insensitive to different scaling factors for AUD base rates that were used to impute missing FH status in the primary analysis (see manuscript section 3.1.1).


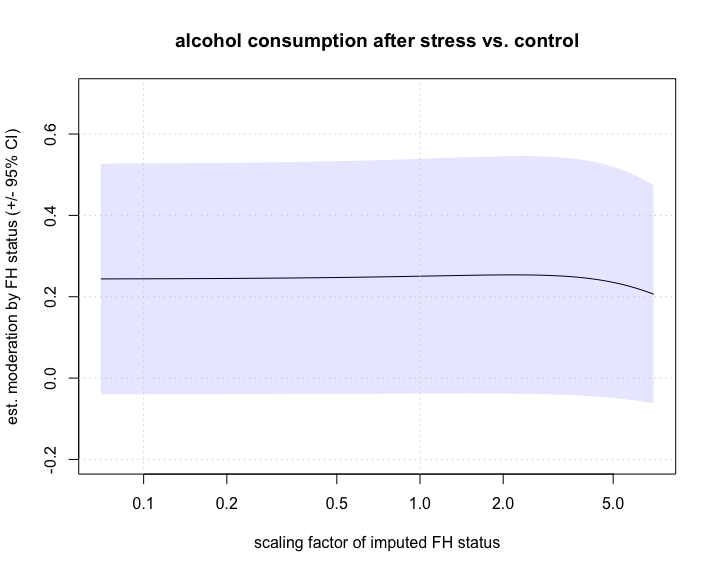


## Meta-analysis of alcohol-related cortisol response

## Based on multivariate meta-regression modelling, acute alcohol consumption did not significantly predict C_MaxMin_ (g_REE_= -0.00 SD, SE= 0.07; CI_95%_= [-0.14, 0.13] and g_PEESE_= 0.01 SD, SE= 0.07; CI_95%_= [-0.13, 0.16]) indicating no substantial change in cortisol *reactivity*. By contrast, effects on C_Min_ suggested a slight decrease of alcohol-related cortisol *recovery* (C_Min_⭡; g_REE_= 0.14 SD, SE= 0.09; CI_95%_= [-0.02, 0.31] and g_PEESE_= 0.16 SD, SE= 0.09; CI_95%_= [-0.02, 0.33]). Similarly, the analysis of C_AUC_ showed a consistent increase after acute alcohol as compared to placebo consumption (g_REE_= 0.38 SD, SE= 0.27; CI_95%_= [-0.15, 0.91] and g_PEESE_= 0.44 SD, SE= 0.28; CI_95%_= [-0.11, 0.99]). The residual heterogeneity of alcohol-related C_MaxMin_ (τ= 0.16), C_Min_ (τ= 0.20), and C_AUC_ (τ= 0.47) was significant (Q= 160.39; p< .001). C_MaxMin_ was positively correlated with C_AUC_ (*r* = 0.80) and negatively correlated with C_Min_ (*r* = -0.61), whereas the latter C_AUC_ and C_Min_ were not estimated to be correlated (r = -0.00).

# Meta-regression of stress-related alcohol consumption on family history of AUD

## FH+ individuals showed an increased alcohol-related cortisol *reactivity* when samples with unknown FH status were not imputed but removed from the analysis set (C_MaxMin_⭡; β= 0.24 SD, SE= 0.09; CI_95%_= [0.06, 0.42]). As shown below, this moderation effect was insensitive to different scaling factors for AUD base rates that were used to impute missing FH status in the primary analysis (see manuscript section 3.2.1).


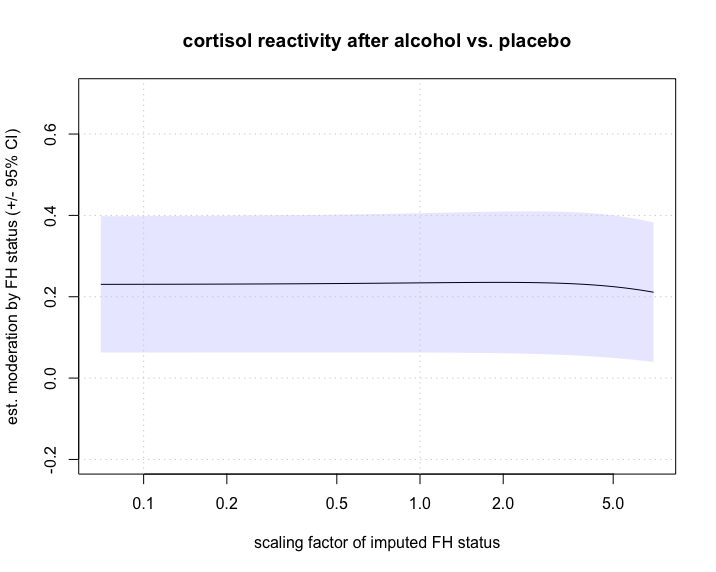


## FH+ individuals showed no considerable different to FH- individuals with regard to alcohol-related cortisol *recovery* when samples with unknown FH status were not imputed but removed from the analysis set (C_Min_; β= -0.03 SD, SE= 0.09; CI_95%_= [-0.20, 0.14]). As shown below, this claim for absence of a pronounced moderation effect was robust across the range of scaling factors for AUD base rates that were used to impute missing FH status in the primary analysis.


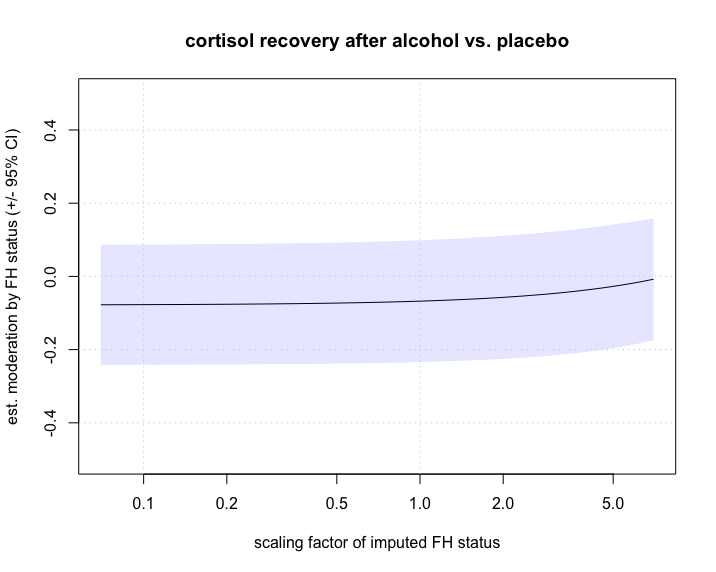


## Meta-regression of alcohol-related cortisol response on acute stress

**Table S1**. Meta-regression of alcohol-related cortisol turnover under concurrent acute stress exposure.

|  | | Cortisol turnover, C_AUC_ | | | | |
| --- | --- | --- | --- | --- | --- | --- |
| **Stratum** | | **k** | **Estimate (SE)** | **[95% CI]** | **Q _M_(df)** | **p value** |
| **Age in years** | | 16 |  | | 0.11(1) | 0.75 |
|  | Ø 20.0 yrs |  | +0.13 (0.87) | [-1.57, 1.83] |  |  |
|  | Ø 25.3 yrs |  | +0.03 (0.59) | [-1.13, 1.18] |  |  |
|  | Ø 33.5 yrs |  | -0.58 (1.47) | [-3.47, 2.30] |  |  |
| **Percent male subjects** | | 16 |  | | 3.42 (1) | 0.06 |
|  | Ø 0% |  | +0.94 (0.67) | [-0.37, 2.24] |  |  |
|  | Ø 100% |  | -0.22 (0.11) | [-0.43, 0.00] |  |  |
| **Comorbidity inclusion** | | 16 |  | | 0.40(1) | 0.53 |
|  | No |  | -0.17 (0.34) | [-0.84, 0.50] |  |  |
|  | Some |  | +0.22 (0.66) | [-1.08, 1.52] |  |  |
| **AUD status** | | 16 |  | | 0.66(1) | 0.42 |
|  | AUD- |  | +0.23 (0.33) | [-0.87, 0.41] |  |  |
|  | AUD+ |  | -0.11 (0.32) | [-0.74, 0.52] |  |  |
| **FH status** | | 16 |  | | 0.22(1) | 0.64 |
|  | Ø 0% FH+ (==FH-) |  | -0.13 (0.32) | [-0.76, 0.50] |  |  |
|  | Ø 10% FH+ |  | -0.13 (0.32) | [-0.76, 0.50] |  |  |
|  | Ø 100% FH+ |  | -0.19 (0.32) | [-0.83, 0.44] |  |  |
| **Dose consumed per kg** | | 16 |  | | 4.35(1) | 0.04 |
|  | Ø 0.1g |  | +0.27 (0.28) | [-0.28, 0.82] |  |  |
|  | Ø 0.5g |  | -0.21 (0.11) | [-0.43, 0.01] |  |  |
|  | Ø 2.0g |  | -1.97 (0.82) | [-3.58, -0.36] |  |  |
| **Stressor type** | | 16 |  |  | 14.82(3) | 0.00 |
|  | No stress exposure | | -0.12 (0.14) | [-0.39, 0.15] |  |  |
|  | Cognitive |  | -0.61 (0.14) | [-0.89, 0.34] |  |  |
|  | Pharmacological (CRH) | | -1.00 (0.43) | [-1.85, -0.14] |  |  |
|  | Social-evaluative |  | -0.65 (0.43) | [-1.50, 0.20] |  |  |

*Note*. k = number of study samples, iv = intravenous, AUD = alcohol use disorder. All estimates are adjusted for the precision of effect estimates (PEESE). Moderator levels that could not be estimated due to unavailable data (e.g., data on alcohol-related cortisol *reactivity* or *recovery* in currently abstinent individuals who previously met AUD criteria AUD±) are not reported. When moderators lacked discrete levels, their minimum, median and maximum values were contrasted. Please note that Table 3 is based on the same set of studies as Table 2 and ten additional studies featuring concurrent acute stress exposure.

# Meta-Analysis of observed cortisol responses

The analysis included n = 44 studies, providing a total of k = 234 effect sizes (C_MaxMin_: 126, C_Min_: 110), and encompassed 2344 individuals with a mean age 19.8 to 53.1 years to explore determinants of stress- or drug-induced cortisol *reactivity* (C_MaxMin_) and *recovery* (C_Min_) in individuals with varying AUD status and across different types of stressors or drugs. 82.04 % were male and 17.96 % were female, 9.81 % were AUD+ individuals, 37.80 % were AUD- individuals, and 52.39 % previously met the criteria but were currently abstinent for a mean minimum duration of 19.93± 16.73 days (AUD± individuals). Family history of AUD was assessed by only two studies and was therefore not further considered.

Using meta-analyses, we estimated the baseline-standardized differences between maximum and minimum cortisol levels (C_MaxMin_) and the baseline-standardized minimum cortisol levels (C_Min_) for each available type of stressor and drug class that was used to induce cortisol responses.

As listed in **Table S2**, cortisol *reactivity* (C_MaxMin_) appeared to be slightly lower in studies that exposed individuals to physiological or cognitive stressors. Those studies investigating AUD± individuals reported, on average, considerably larger cortisol *reactivity* (C_MaxMin_⭡; and negligibly reduced cortisol *recovery*, C_Min_⭡) than studies investigating AUD- individuals. The only study investigating AUD+ individuals reported, on average, fifty percent lower cortisol *reactivity* (C_MaxMin_⭣) than those studies investigating AUD- individuals^L7^. As listed in **Table S3**, a similar picture arises across different classes of potentially cortisol-affective drugs. That is, AUD± individuals demonstrate larger cortisol *reactivity* (C_MaxMin_⭡), whereas AUD+ individuals demonstrate lower cortisol *reactivity* (C_MaxMin_⭣). The highest cortisol *reactivity (*C_MaxMin_⭡) is observable in response to synthetic cortisol administration (or direct stimulants) and direct suppressors.

To finally explore whether the minimum duration of abstinence before study inclusion predicts cortisol *reactivity* (C_MaxMin_) and *recovery* (C_Min_) in AUD± individuals, we conducted a separate multi-level meta-regression of stress- or drug-induced cortisol *reactivity* (C_MaxMin_) and *recovery* (C_Min_) on this minimum duration of abstinence in days in the (sub-)samples of the respective studies. As could be naively inferred from supplementary **Figure S2**, stress-induced increases in cortisol *reactivity* (C_MaxMin_⭡) seem to be associated with increasing duration of abstinence and drug-induced decreases in cortisol *recovery* (C_Min_⭡) seem to be associated with increasing duration of abstinence. However, those associations are not robust, but driven by between-study heterogeneity (and small study effects) and thus constitute an illustration of Simpson’s paradox.

**Table S2**. Meta-analysis of stress-induced cortisol *reactivity* (C_MaxMin_, difference between maximum and minimum levels) and *recovery* (C_Min_, minimum levels), stratified by stressor type and AUD status as potential determinants of cortisol measures.

| Stressor | AUD-  individuals with no AUD diagnosis | | | | | AUD+  actively drinking individuals meeting AUD criteria | | | | | AUD±  abstinent individuals who previously met AUD criteria | | | | |
| --- | --- | --- | --- | --- | --- | --- | --- | --- | --- | --- | --- | --- | --- | --- | --- |
|  | k | g_REE_ | SE | CI_2.5%_ | CI_97.5%_ | k | g_REE_ | SE | CI_2.5%_ | CI_97.5%_ | k | g_REE_ | SE | CI_2.5%_ | CI_97.5%_ |
| C_MaxMin_ |  |  |  |  |  |  |  |  |  |  |  |  |  |  |  |
| Control | 7 | 2.57 | 1.12 | 0.37 | 4.77 | 1 | 1.47 | 0.25 | 0.98 | 1.97 | 4 | 2.92 | 0.66 | 1.62 | 4.21 |
| Cognitive | 5 | 1.39 | 0.16 | 1.06 | 1.71 | 0 | - | - | - | - | 5 | 1.71 | 0.35 | 1.03 | 2.39 |
| Imagery | 5 | 2.01 | 0.65 | 0.74 | 3.28 | 0 | - | - | - | - | 2 | 3.53 | 0.69 | 2.17 | 4.88 |
| Physiologic | 4 | 0.93 | 0.16 | 0.61 | 1.24 | 1 | 2.26 | 0.34 | 1.60 | 2.92 | 2 | 1.84 | 0.29 | 1.28 | 2.40 |
| Social | 10 | 1.84 | 0.91 | 0.05 | 3.62 | 2 | 1.38 | 0.87 | -0.33 | 3.09 | 12 | 2.43 | 0.76 | 0.94 | 3.91 |
| C_Min_ |  |  |  |  |  |  |  |  |  |  |  |  |  |  |  |
| Control | 4 | 3.94 | 1.55 | 0.89 | 6.99 | 1 | 1.03 | 0.19 | 0.65 | 1.41 | 3 | 3.35 | 1.55 | 0.32 | 6.38 |
| Cognitive | 5 | 2.23 | 0.24 | 1.76 | 2.70 | 0 | - | - | - | - | 5 | 2.67 | 0.41 | 1.87 | 3.46 |
| Imagery | 2 | 2.95 | 0.29 | 2.38 | 3.53 | 0 | - | - | - | - | 0 | - | - | - | - |
| Physiologic | 4 | 4.03 | 0.28 | 3.47 | 4.59 | 0 | - | - | - | - | 2 | 4.30 | 1.34 | 1.67 | 6.93 |
| Social | 8 | 3.19 | 1.42 | 0.40 | 5.98 | 2 | 1.88 | 0.09 | 1.71 | 2.05 | 11 | 2.77 | 0.92 | 0.96 | 4.57 |

**Table S3**. Meta-analysis of stress-induced cortisol *reactivity* (C_MaxMin_, difference between maximum and minimum levels) and *recovery* (C_Min_, minimum levels), stratified by drug class and AUD status as potential determinants of cortisol measures. We classified the following drugs as cortisol-stimulants: synthetic Adrenocorticotropin (ACTH) Cosyntropin, human or ovine Corticotropin-Releasing-Hormon (CRH), dexamethosaon+CRH, synthetic cortisol hydrocortisone; as direct cortisol-supressors: dexamethasone; indirect cortisol-stimulant: Naloxone, naltrexone, yohimbine, busiprone, Meta-Chlorophenylpiperazine (MCP), insulin, ghrelin, glucose.

| Drug Class | AUD-  individuals with no AUD diagnosis | | | | | AUD+  actively drinking individuals meeting AUD criteria | | | | | AUD±  abstinent individuals who previously met AUD criteria | | | | |
| --- | --- | --- | --- | --- | --- | --- | --- | --- | --- | --- | --- | --- | --- | --- | --- |
|  | k | g_REE_ | SE | CI_2.5%_ | CI_97.5%_ | k | g_REE_ | SE | CI_2.5%_ | CI_97.5%_ | k | g_REE_ | SE | CI_2.5%_ | CI_97.5%_ |
| C_MaxMin_ |  |  |  |  |  |  |  |  |  |  |  |  |  |  |  |
| Placebo | 5 | 0.89 | 0.25 | 0.40 | 1.37 | 3 | 0.63 | 0.11 | 0.42 | 0.85 | 2 | 0.54 | 0.10 | 0.35 | 0.73 |
| Direct cortisol-stimulants | 17 | 2.85 | 0.58 | 1.73 | 3.98 | 0 | - | - | - | - | 9 | 7.32 | 2.41 | 2.59 | 12.05 |
| Direct cortisol-suppressors | 4 | 7.12 | 3.05 | 1.13 | 13.10 | 0 | - | - | - | - | 2 | 12.89 | 7.12 | -1.06 | 26.85 |
| Indirect cortisol-stimulants | 12 | 1.61 | 0.26 | 1.10 | 2.13 | 3 | 0.88 | 0.14 | 0.61 | 1.15 | 7 | 2.19 | 0.36 | 1.48 | 2.89 |
| C_Min_ |  |  |  |  |  |  |  |  |  |  |  |  |  |  |  |
| Placebo | 5 | 2.51 | 0.43 | 1.68 | 3.35 | 3 | 1.44 | 0.11 | 1.22 | 1.65 | 2 | 2.92 | 0.08 | 2.75 | 3.08 |
| Direct cortisol-stimulants | 16 | 1.64 | 0.19 | 1.25 | 2.02 | 0 | - | - | - | - | 9 | 3.38 | 1.17 | 1.09 | 5.67 |
| Direct cortisol-suppressors | 4 | 1.69 | 0.30 | 1.10 | 2.27 | 0 | 1.70 | 0.23 | 1.24 | 2.15 | 2 | 2.11 | 0.53 | 1.06 | 3.15 |
| Indirect cortisol-stimulants | 12 | 2.71 | 0.21 | 2.31 | 3.12 | 3 | 2.96 | 0.34 | 2.29 | 3.64 | 7 | 2.96 | 0.34 | 2.29 | 3.64 |

.

# Supplementary Figures

A

A

**Figure S1**. Contour-enhanced funnel plots for study effect estimates on alcohol-related cortisol responses as indicated by (**Upper Left**) differences between the maximum and minimum cortisol levels (C_MaxMin_), (**Upper Right**) the minimum cortisol levels (C_Min_) and (**Lower Left**) cortisol concentration-time curves (C_AUC_). (**Lower Right**) Forest plot of the reported and estimated effects of acute alcohol exposure on cortisol concentration-time curves (C_AUC_). The dotted vertical line signifies the scenario of “no efficacy of acute alcohol consumption”. Square symbols indicate the effect size reported by the respective sample, with their size indicating relative number of individuals informing that sample. Error bars indicate 95% confidence interval (CI) around each estimate. Diamond symbols indicate meta-analytical effect estimates. REE: naïve random-effects estimate, PEESE: precision effect estimate with standard error. AUD=Alcohol use disorder; FH= Family history of alcohol consumption; + =active/positive; -=absent/negative.

**Figure S2.** Meta-regression of stress and drug-induced alterations of cortisol *reactivity* (C_MaxMin_; baseline-standardized differences between maximum and minimum cortisol levels) and *recovery* (C_Min_; baseline-standardized minimum cortisol levels) by the minimum duration of abstinence in days in abstinent individuals who previously met AUD criteria (AUD±). REE= naïve random-effects estimate, PEESE= precision effect estimate with standard error. The size of circles indicate the relative number of individuals informing the respective study (sub-)sample, whereas the text label indicates the study ID. The red dots represent study (sub-)samples that were comprised of actively drinking individuals with AUD (AUD+, hence their minimum duration of abstinence = 0 days).


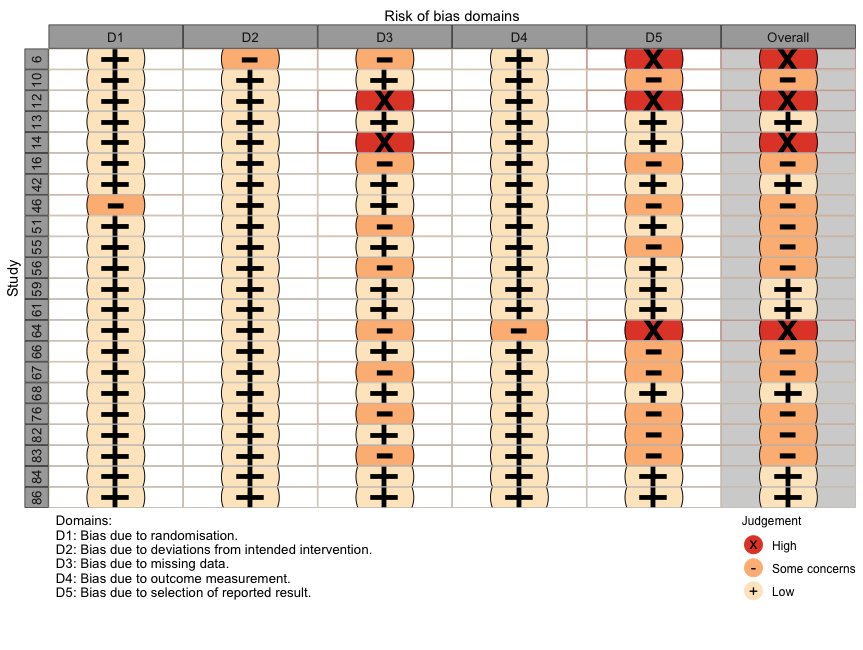


**Figure S3.** Traffic light plot to assess the risk of bias in the 20 studies informing the “Meta-Analysis of Stress-Related Alcohol Consumption”. Study 6:Bacon 2013, 10:Bernstein 2017, 12:Brkic 2015, 13:Buckheit 2023, 14:Caudill 1975, 16:Clay 2018, 42:Hull 1983, 46:Kidorf 1999, 51:Laessle 2016, 55:Magrys 2015, 56:Marlatt 1975, 59:McGrath 2016, 61:Miller 1974, 64:Nesic 2006, 66:Patock-Peckham 2022, 67:Pihl 1994, 68:Pratt 2009, 76:Soederpalm Gordh 2011, 82:Talley 2022, 83:Thomas 2011, 84:Thomas 2014, 86:Tucker 1980


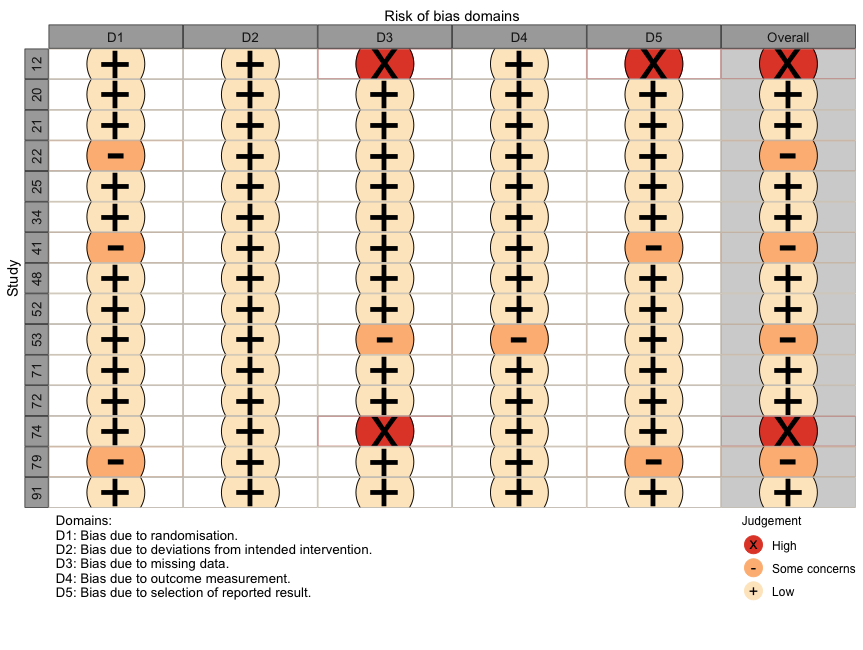


**Figure S4.** Traffic light plot to assess the risk of bias in the 15 studies informing the “Meta-Analysis of Alcohol-Related Cortisol Response”. Study 12:Brkic 2015, 20:Dai 2002, 21:Dai 2007, 22:Davis 1983, 25:Dolinsky 1987, 34:Gianoulakis 1989, 41:Ho 1988, 48:King 2006, 52:Lex 1991, 53:Linkola 1979, 71:Sarkola 1999, 72:Schuckit 1987, 74:Seto 1978, 79:Soyka 1991, 91:Wall 1994

# Reference list of meta-analysed studies

L1 Adinoff, B., Leonard, D., Price, J., Javors, M. A., Walker, R., Brown, E. S., et al. Adrenocortical sensitivity, moderated by ongoing stress, predicts drinking intensity in alcohol-dependent men. Psychoneuroendocrinology. 2017; 76: 67–76.

L2 Anthenelli, R. M., Heffner, J. L., Blom, T. J., Daniel, B. E., McKenna, B. S., Wand, G. S. Sex differences in the ACTH and cortisol response to pharmacological probes are stressor-specific and occur regardless of alcohol dependence history. Psychoneuroendocrinology. 2018; 94: 72–82.

L3 Bach, P., Zaiser, J., Zimmermann, S., Gessner, T., Hoffmann, S., Gerhardt, S., et al. Stress-induced sensitization of insula activation predicts alcohol craving and alcohol use in alcohol use disorder. Biol Psychiatry. 2024; 95: 245–255.

L4 Bailly, D., Dewailly, D., Beuscart, R., Couplet, G., Dumont, P., Racadot, A., et al. Adrenocorticotropin and cortisol responses to ovine corticotropin-releasing factor in alcohol dependence disorder: preliminary report. Hormones. 1989; 31: 72–75.

L5 Balodis, I. M., Wynne-Edwards, K. E., Olmstead, M. C. The stress–response-dampening effects of placebo. Horm Behav. 2011; 59: 465–472.

L6 Bernstein, M. H., Wood, M. D. Effect of anticipatory stress on placebo alcohol consumption in a bar laboratory. Am J Drug Alcohol Abuse. 2017; 43: 95–102.

L7 Bibbey, A., Phillips, A. C., Ginty, A. T., Carroll, D. Problematic Internet use, excessive alcohol consumption, their comorbidity and cardiovascular and cortisol reactions to acute psychological stress in a student population. J Behav Addict. 2015; 4: 44–52.

L8 Blaine, S. K., Nautiyal, N., Hart, R., Guarnaccia, J. B., Sinha, R. Craving, cortisol and behavioral alcohol motivation responses to stress and alcohol cue contexts and discrete cues in binge and non-binge drinkers. Addict Biol. 2019; 24: 1096–1108.

L9 Brkic, S., Söderpalm, B., Gordh, A. S. A family history of Type 1 alcoholism differentiates alcohol consumption in high cortisol responders to stress. Pharmacol Biochem Behav. 2015; 130: 59–66.

L10 Buckheit, K. A., Maisto, S. A. Stress and alcohol use: An experimental investigation of cognitive mechanisms. Addict Neurosci. 2023; 6: 100078.

L11 Childs, E., O’Connor, S., de Wit, H. Bidirectional interactions between acute psychosocial stress and acute intravenous alcohol in healthy men. Alcohol Clin Exp Res. 2011; 35: 1794–1803.

L12 Clay, J. M., Parker, M. O. The role of stress-reactivity, stress-recovery and risky decision-making in psychosocial stress-induced alcohol consumption in social drinkers. Psychopharmacology. 2018; 235: 3243–3257.

L13 Coiro, V., Casti, A., Saccani Jotti, G., Rubino, P., Manfredi, G., Maffei, M. L., et al. Adrenocorticotropic hormone/cortisol response to physical exercise in abstinent alcoholic patients. Alcohol Clin Exp Res. 2007; 31: 901–906.

L14 Costa, A., Bono, G., Martignoni, E., Merlo, P., Sances, G., Nappi, G. An assessment of hypothalamo-pituitary-adrenal axis functioning in non-depressed, early abstinent alcoholics. Psychoneuroendocrinology. 1996; 21: 263–275.

L15 Croissant, B., Rist, F., Demmel, R., Olbrich, R. Alcohol-induced heart rate response dampening during aversive and rewarding stress paradigms in subjects at risk for alcoholism. Int J Psychophysiol. 2006; 61: 253–261.

L16 Dai, X., Thavundayil, J., Santella, S., Gianoulakis, C. Response of the HPA-axis to alcohol and stress as a function of alcohol dependence and family history of alcoholism. Psychoneuroendocrinology. 2007; 32: 293–305.

L17 Davis, J. R. E., Jeffcoate, W. J. Lack of effect of ethanol on plasma cortisol in man. Clin Endocrinol. 1983; 19: 461–466.

L18 de Wit, H., Söderpalm, A. H., Nikolayev, L., Young, E. Effects of acute social stress on alcohol consumption in healthy subjects. Alcohol Clin Exp Res. 2003; 27: 1270–1277.

L19 de Wit, M., Wiaterek, G. K., Gray, N. D., et al. Relationship between alcohol use disorders, cortisol concentrations, and cytokine levels in patients with sepsis. Crit Care. 2010; 14: R230.

L20 Ehrenreich, H., Schuck, J., Stender, N., Pilz, J., Gefeller, O., Schilling, L., et al. Endocrine and hemodynamic effects of stress versus systemic CRF in alcoholics during early and medium term abstinence. Alcohol Clin Exp Res. 1997; 21: 1285–1293.

L21 Eisenhofer, G., Johnson, R. H., Lambie, D. G. Growth hormone, vasopressin, cortisol, and catecholamine responses to insulin hypoglycemia in alcoholics. Alcohol Clin Exp Res. 1984; 8: 33–36.

L22 Errico, A. L., King, A. C., Lovallo, W. R., Parsons, O. A. Cortisol dysregulation and cognitive impairment in abstinent male alcoholics. Alcohol Clin Exp Res. 2002; 26: 1198–1204.

L23 Errico, A. L., Parsons, O. A., King, A. C., Lovallo, W. R. Attenuated cortisol response to biobehavioral stressors in sober alcoholics. J Stud Alcohol. 1993; 54: 393–398.

L24 Fink, R. S., Short, F., Marjot, D. H., James, V. H. T. Abnormal suppression of plasma cortisol during the intravenous infusion of dexamethasone to alcoholic patients. Clin Endocrinol. 1981; 15: 97–102.

L25 Fox, H. C., Bergquist, K. L., Hong, K. I., Sinha, R. Stress-induced and alcohol cue-induced craving in recently abstinent alcohol-dependent individuals. Alcohol Clin Exp Res. 2007; 31: 395–403.

L26 Fox, H. C., Anderson, G. M., Tuit, K., Hansen, J., Kimmerling, A., Siedlarz, K. M., et al. Prazosin effects on stress- and cue-induced craving and stress response in alcohol-dependent individuals: preliminary findings. Alcohol Clin Exp Res. 2012; 36: 351–360.

L27 Gianoulakis, C., Beliveau, D., Angelogianni, P., Meaney, M., Thavundayil, J., Tawar, V., et al. Different pituitary β-endorphin and adrenal cortisol response to ethanol in individuals with high and low risk for future development of alcoholism. Life Sci. 1989; 45: 1097–1109.

L28 Haass-Koffler, C. L., Long, V. M., Farokhnia, M., Magill, M., Kenna, G. A., Swift, R. M., et al. Intravenous administration of ghrelin increases serum cortisol and aldosterone concentrations in heavy-drinking alcohol-dependent individuals: results from a double-blind, placebo-controlled human laboratory study. Neuropharmacology. 2019; 158: 107711.

L29 Haass-Koffler, C. L., Magill, M., Cannella, N., Brown, J. C., Aoun, E. G., Cioe, P. A., et al. Mifepristone as a pharmacological intervention for stress-induced alcohol craving: a human laboratory study. Addict Biol. 2023; 28: e13288.

L30 Haugvad, A., Haugvad, L., Hamarsland, H., Paulsen, G. Ethanol does not delay muscle recovery but decreases testosterone/cortisol ratio. Med Sci Sports Exerc. 2014; 46: 2175–2183.

L31 Haxholdt, O. S., Johansson, G. The alcoholic patient and surgical stress. Anaesthesia. 1982; 37: 797–801.

L32 Heikkonen, E., Ylikahri, R., Roine, R., Välimäki, M., Härkönen, M., Salaspuro, M. The combined effect of alcohol and physical exercise on serum testosterone, luteinizing hormone, and cortisol in males. Alcohol Clin Exp Res. 1996; 20: 711–716.

L33 Heuser, I., von Bardeleben, U., Boll, E., Holsboer, F. Response of ACTH and cortisol to human corticotropin-releasing hormone after short-term abstention from alcohol abuse. Biol Psychiatry. 1988; 24: 316–321.

L34 Higgins, R. L., Marlatt, G. A. Effects of anxiety arousal on the consumption of alcohol by alcoholics and social drinkers. J Consult Clin Psychol. 1973; 41: 426.

L35 Ho, S. B., DeMaster, E. G., Shafer, R. B., Levine, A. S., Morley, J. E., Go, V. L. W., et al. Opiate antagonist nalmefene inhibits ethanol-induced flushing in Asians: a preliminary study. Alcohol Clin Exp Res. 1988; 12: 705–712.

L36 Hundt, W., Zimmermann, U., Pöttig, M., Spring, K., Holsboer, F. The combined dexamethasone-suppression/CRH-stimulation test in alcoholics during and after acute withdrawal. Alcohol Clin Exp Res. 2001; 25: 687–691.

L37 Kemper, A., Koalick, F., Thiele, H., Retzow, A., Rathsack, R., Nickel, B. Cortisol and β-endorphin response in alcoholics and alcohol abusers following a high naloxone dosage. Drug Alcohol Depend. 1990; 25: 319–326.

L38 Kidorf, M., Lang, A. R. Effects of social anxiety and alcohol expectancies on stress-induced drinking. Psychol Addict Behav. 1999; 13: 134–142.

L39 Kim, Y. H., Shim, J. C., Kelly, D. L., Lee, J. G., Seo, Y. S., Conley, R. R. Cortisol response to buspirone in extended abstinent alcoholics. Alcohol Alcohol. 2004; 39: 287–289.

L40 King, A., Munisamy, G., de Wit, H., Lin, S. Attenuated cortisol response to alcohol in heavy social drinkers. Int J Psychophysiol. 2006; 59: 203–209.

L41 Knudsen, G. M., Christensen, H., Berild, D., Melgaard, B., Kirkegaard, C., Hasselbalch, H. Discordance between the cortisol response to insulin-hypoglycemia and 30-minute ACTH stimulation test in chronic alcoholic men. Alcohol Clin Exp Res. 1987; 11: 323–325.

L42 Kwako, L. E., Schwandt, M. L., Sells, J. R., Ramchandani, V. A., Hommer, D. W., George, D. T., et al. Methods for inducing alcohol craving in individuals with co-morbid alcohol dependence and posttraumatic stress disorder: Behavioral and physiological outcomes. Addict Biol. 2015; 20: 733–746.

L43 Laessle, R. G., Preuss, A. Stressinduzierter Alkoholkonsum bei Frauen und Männern – eine Laborstudie [Stress induced alcohol drinking in men and women – A laboratory experiment]. Verhaltenstherapie und Verhaltensmedizin. 2016; 37: 417–421.

L44 Lex, B. W., Ellingboe, J. E., Teoh, S. K., Mendelson, J. H., Rhoades, E. Prolactin and cortisol levels following acute alcohol challenges in women with and without a family history of alcoholism. Alcohol. 1991; 8: 383–387.

L45 Linkola, J., Fyhrquist, F., Ylikahri, R. Renin, aldosterone and cortisol during ethanol intoxication and hangover. Acta Physiol Scand. 1979; 106: 75–82.

L46 Lovallo, W. R., Dickensheets, S. L., Myers, D. A., Thomas, T. L., Nixon, S. J. Blunted stress cortisol response in abstinent alcoholic and polysubstance‐abusing men. Alcohol Clin Exp Res. 2000; 24: 651–658.

L47 Magrys, S. A., Olmstead, M. C. Acute stress increases voluntary consumption of alcohol in undergraduates. Alcohol Alcohol. 2015; 50: 213–218.

L48 Marlatt, G. A., Kosturn, C. F., Lang, A. R. Provocation to anger and opportunity for retaliation as determinants of alcohol consumption in social drinkers. J Abnorm Psychol. 1975; 84: 652–661.

L49 Marra, D., Warot, D., Berlin, I., Hispard, E., Dally, S. Psychological stress and cortisol secretion in anhedonic alcoholic men. Eur Psychiatry. 2003; 18: 415–417.

L50 McGrath, E., Jones, A., Field, M. Acute stress increases ad-libitum alcohol consumption in heavy drinkers, but not through impaired inhibitory control. Psychopharmacology. 2016; 233: 1227–1234.

L51 Meng, D., Wu, T., Rao, U., North, C. S., Xiao, H., Javors, M. A., et al. Serum NPY and BNDF response to a behavioral stressor in alcohol-dependent and healthy control participants. Psychopharmacology. 2011; 218: 59–67.

L52 Milivojevic, V., Angarita, G. A., Hermes, G., Sinha, R., Fox, H. C. Effects of prazosin on provoked alcohol craving and autonomic and neuroendocrine response to stress in alcohol use disorder. Alcohol Clin Exp Res. 2020; 44: 1488–1496.

L53 Miller, P. M., Hersen, M., Eisler, R. M., Hilsman, G. Effects of social stress on operant drinking of alcoholics and social drinkers. Behav Res Ther. 1974; 12: 67–72.

L54 Muehlhan, M., Höcker, A., Miller, R., Trautmann, S., Wiedemann, K., Lotzin, A., et al. HPA axis stress reactivity and hair cortisol concentrations in recently detoxified alcoholics and healthy controls with and without childhood maltreatment. Addict Biol. 2020; 25: e12681.

L55 Nakajima, M., Kumar, S., Wittmers, L., Scott, M. S., al’Absi, M. Psychophysiological responses to stress following alcohol intake in social drinkers who are at risk of hazardous drinking. Biol Psychol. 2013; 93: 9–16.

L56 Ozsoy, S., Esel, E. Hypothalamic–pituitary–adrenal axis activity, dehydroepiandrosterone sulphate and their relationships with aggression in early and late alcohol withdrawal. Prog Neuropsychopharmacol Biol Psychiatry. 2008; 32: 340–347.

L57 Patock-Peckham, J. A., Corbin, W. R., Smyth, H., Canning, J. R., Ruof, A., Williams, J. Effects of stress, alcohol prime dose, and sex on ad libitum drinking. Psychol Addict Behav. 2022; 36: 871.

L58 Pratt, W. M., Davidson, D. Role of the HPA axis and the A118G polymorphism of the μ-opioid receptor in stress-induced drinking behavior. Alcohol Alcohol. 2009; 44: 358–365.

L59 Roine, R., Luurila, O. J., Suokas, A., Heikkonen, E., Koskinen, P., Ylikahri, R., et al. Alcohol and sauna bathing: effects on cardiac rhythm, blood pressure, and serum electrolyte and cortisol concentrations. J Intern Med. 1992; 231: 333–338.

L60 Schuckit, M. A., Gold, E., Risch, C. Plasma cortisol levels following ethanol in sons of alcoholics and controls. Arch Gen Psychiatry. 1987; 44: 942–945.

L61 Schwarze, Y., Voges, J., Schröder, A., Dreeßen, S., Voß, O., Krach, S., et al. Altered physiological, affective, and functional connectivity responses to acute stress in patients with alcohol use disorder. Biol Psychiatry Glob Open Sci. 2024; 4: 100358.

L62 Sinha, R., Fox, H. C., Hong, K. A., Bergquist, K., Bhagwagar, Z., Siedlarz, K. M. Enhanced negative emotion and alcohol craving, and altered physiological responses following stress and cue exposure in alcohol dependent individuals. Neuropsychopharmacology. 2009; 34: 1198–1208.

L63 Sinha, R., Fox, H. C., Hong, K. I. A., Hansen, J., Tuit, K., Kreek, M. J. Effects of adrenal sensitivity, stress- and cue-induced craving, and anxiety on subsequent alcohol relapse and treatment outcomes. Arch Gen Psychiatry. 2011; 68: 942–952.

L64 Söderpalm, A. H., de Wit, H. Effects of stress and alcohol on subjective state in humans. Alcohol Clin Exp Res. 2002; 26: 818–826.

L65 Söderpalm Gordh, A. H., Brkic, S., Söderpalm, B. Stress and consumption of alcohol in humans with a Type 1 family history of alcoholism in an experimental laboratory setting. Pharmacol Biochem Behav. 2011; 99: 696–703.

L66 Soravia, L. M., Moggi, F., de Quervain, D. J. F. Effects of cortisol administration on craving during in vivo exposure in patients with alcohol use disorder. Transl Psychiatry. 2021; 11: 6.

L67 Soyka, M., Görig, E., Naber, D. Serum prolactin increase induced by ethanol—a dose-dependent effect not related to stress. Psychoneuroendocrinology. 1991; 16: 441–446.

L68 Starcke, K., van Holst, R. J., van den Brink, W., Veltman, D. J., Goudriaan, A. E. Physiological and endocrine reactions to psychosocial stress in alcohol use disorders: duration of abstinence matters. Alcohol Clin Exp Res. 2013; 37: 1343–1350.

L69 Talley, A. E., Harris, B. N., Le, T. H., Hohman, Z. P. Aversive self-focus and alcohol consumption behavior in women with sexual identity-uncertainty: changes in salivary cortisol stress response among those who drink-to-cope. Chronic Stress. 2022.

L70 Thomas, S. E., Randall, P. K., Brady, K., See, R. E., Drobes, D. J. An acute psychosocial stressor does not potentiate alcohol cue reactivity in non-treatment-seeking alcoholics. Alcohol Clin Exp Res. 2011; 35: 464–473.

L71 Thomas, S. E., Merrill, J. E., von Hofe, J., Magid, V. Coping motives for drinking affect stress reactivity but not alcohol consumption in a clinical laboratory setting. J Stud Alcohol Drugs. 2014; 75: 115–123.

L72 Tolic, I., Soyka, M. Stress response in persons with alcohol addiction in the context of abstinence duration and disease severity. Fortschr Neurol Psychiatr. 2018; 86: 356–367.

L73 Tacker, J. A., Vuchinich, R. E., Sobell, M. B., Maisto, S. A. Normal drinkers' alcohol consumption as a function of conflicting motives induced by intellectual performance stress. Addict Behav. 1980; 5: 171–178.

L74 Uhart, M., Oswald, L., McCaul, M. E., Chong, R., Wand, G. S. Hormonal responses to psychological stress and family history of alcoholism. Neuropsychopharmacology. 2006; 31: 2255–2263.

L75 Umhau, J. C., Schwandt, M. L., Usala, J., Geyer, C., Singley, E., George, D. T., et al. Pharmacologically induced alcohol craving in treatment seeking alcoholics correlates with alcoholism severity, but is insensitive to acamprosate. Neuropsychopharmacology. 2011; 36: 1178–1186.

L76 Vescovi, P. P., DiGennaro, C., Coiro, V. Hormonal (ACTH, cortisol, β‐endorphin, and met‐enkephalin) and cardiovascular responses to hyperthermic stress in chronic alcoholics. Alcohol Clin Exp Res. 1997; 21: 1195–1198.

L77 von Bardeleben, U., Heuser, I., Holsboer, F. Human CRH stimulation response during acute withdrawal and after medium-term abstention from alcohol abuse. Psychoneuroendocrinology. 1989; 14: 441–449.

L78 Wall, T. L., Nemeroff, C. B., Ritchie, J. C., Ehlers, C. L. Cortisol responses following placebo and alcohol in Asians with different ALDH2 genotypes. J Stud Alcohol. 1994; 55: 207–213.

L79 Waltman, C., McCaul, M. E., Wand, G. S. Adrenocorticotropin responses following administration of ethanol and ovine corticotropin-releasing hormone in the sons of alcoholics and control subjects. Alcohol Clin Exp Res. 1994; 18: 826–830.

L80 Wand, G. S., Weerts, E. M., Kuwabara, H., Wong, D. F., Xu, X., McCaul, M. E. The relationship between naloxone-induced cortisol and mu opioid receptor availability in mesolimbic structures is disrupted in alcohol dependent subjects. Alcohol. 2012; 46: 511–517.

L81 Wand, G. S., Weerts, E. M., Kuwabara, H., Wong, D. F., Xu, X., McCaul, M. E., et al. The relationship between naloxone-induced cortisol and delta opioid receptor availability in mesolimbic structures is disrupted in alcohol-dependent subjects. Addict Biol. 2013; 18: 181–192.

L82 Wyckmans, F., Chatard, A., Kornreich, C., Gruson, D., Jaafari, N., Noël, X. Impact of provoked stress on model-free and model-based reinforcement learning in individuals with alcohol use disorder. Addict Behav Rep. 2024; 20: 100574.

L83 Zhang, A., Price, J. L., Leonard, D., North, C. S., Suris, A., Javors, M. A., et al. Alcohol use disorder masks the effects of childhood adversity, lifetime trauma, and chronic stress on hypothalamic–pituitary–adrenal axis reactivity. Alcohol Clin Exp Res. 2020; 44: 1192–1203.

L84 Zimmermann, U., Spring, K., Kunz-Ebrecht, S. R., Uhr, M., Wittchen, H. U., Holsboer, F. Effect of ethanol on hypothalamic–pituitary–adrenal system response to psychosocial stress in sons of alcohol-dependent fathers. Neuropsychopharmacology. 2004; 29: 1156–1165.
